# FHIT Suppresses Epithelial-Mesenchymal Transition (EMT) and Metastasis in Lung Cancer through Modulation of MicroRNAs

**DOI:** 10.1371/journal.pgen.1004652

**Published:** 2014-10-23

**Authors:** Sung-Suk Suh, Ji Young Yoo, Ri Cui, Balveen Kaur, Kay Huebner, Taek-Kyun Lee, Rami I. Aqeilan, Carlo M. Croce

**Affiliations:** 1 Department of Molecular Virology, Immunology and Medical Genetics, and Comprehensive Cancer Center, Ohio State University, Columbus, Ohio, United States of America; 2 South Sea Environmental Research Division, Korea Institute of Ocean Science and Technology, Geoje, South Korea; 3 Dardinger Laboratory for Neuro-oncology and Neurosciences, Department of Neurological Surgery, Comprehensive Cancer Center and The Ohio State University Medical Center, Columbus, Ohio, United States of America; 4 The Lautenberg Center for General and Tumor Immunology, Department of Immunology and Cancer Research, IMRIC, Hebrew University-Hadassah Medical School, Jerusalem, Israel; University of Washington, United States of America

## Abstract

Metastasis is the principal cause of cancer death and occurs through multiple, complex processes that involve the concerted action of many genes. A number of studies have indicated that the Fragile Histidine Triad (*FHIT*) gene product, FHIT, functions as a tumor suppressor in a variety of common human cancers. Although there are suggestions of a role for FHIT loss in progression of various cancers, a role for such loss in metastasis has not been defined. Here, via *in vivo* and *in vitro* assays, we reveal that the enforced expression of FHIT significantly suppresses metastasis, accompanied by inhibition of the epithelial-mesenchymal transition (EMT), a process involved in metastasis through coordinate modulation of EMT-related genes. Specifically, miR-30c, a FHIT-upregulated microRNA, contributes to FHIT function in suppression of EMT and metastasis by directly targeting metastasis genes Metadherin (*MTDH*), High-mobility group AT—hook 2 (*HMGA2*), and the mesenchymal markers, Vimentin (*VIM*) and Fibronectin (*FN1*), in human lung cancer. Finally, we demonstrate that the expression pattern of FHIT and miR-30c is inversely correlated with that of MTDH and HMGA2 in normal tissue, non-metastatic and metastatic tumors, serving as a potential biomarker for metastasis in lung cancer.

## Author Summary

Although Fragile Histidine Triad (*FHIT*) is known as a potential tumor suppressor gene in terms of tumor initiation and progression, the role of FHIT in the metastatic process is not well characterized. Here it is shown that FHIT reduces the motility and invasiveness of lung cancer cells *in vitro* and ability to metastasize *in vivo*, at least partially through the miR-30c-mediated suppression of EMT, a critical process during tumor metastasis. This study provides new insights into the role of FHIT and a FHIT-activated miRNA, miR-30c, as crucial modulators in lung metastasis.

## Introduction

Metastasis, the spread of cells from the site of a primary tumor followed by growth of a secondary tumor in distant organs, is frequently a final and fatal step in tumor progression. This process consists of a series of steps, including angiogenesis, intravasation, survival in circulation, extravasation into surrounding tissue, and formation of micrometastases [1], [2]. In particular, the epithelial-mesenchymal transition (EMT), a process in which epithelial cells lose or modify their apical-basal polarity and are converted to a mesenchymal phenotype, is a critical process during embryonic development and tumor metastasis [3], [4]. In the metastatic process, EMT disrupts the intercellular tight junctions and promotes migration, and self-renewing and stem-like properties to facilitate metastatic colonization [5]. EMT is commonly characterized by the suppression of the cell-cell adhesion receptor E-cadherin and endows cells with more motile, invasive properties [6]. In addition, accumulating evidence from experimental and clinical studies suggests that microRNAs (miRNAs), small, endogenous non-coding RNAs, are involved in EMT. For example, several miRNAs such as miR-200, miR-205 and miR-34 regulate the expression of crucial EMT-related transcription factors, including those belonging to the Snail family, ZEB1, ZEB2 and Twist1/2, by binding to the 3′untranslated regions (UTRs) of their mRNAs [7], [8]. miRNAs have also been shown to promote or suppress various steps in migration and metastasis by directly modulating the expression of metastasis-related genes including *BRMS1*, *PTEN*, *MYC*, and *HMGA2* [9], [10], [11], [12]. Many studies have shown that genes, including miRNA genes, that localize to genome fragile sites or aberrant chromosome regions are associated with cancer, indicating that these breakpoint-associated genes might be important in the development and progression of human cancer [13], [14]. The Fragile Histidine Triad (*FHIT*) gene spans the FRA3B locus on chromosome 3p14.2 and alterations at this common fragile site are associated with many types of cancer [15], [16], [17]. In addition, FHIT is a tumor suppressor that regulates a wide spectrum of biological processes associated with tumor initiation and progression, and FHIT expression is lost in many cancers [18], [19]. Recently, it has been shown that FHIT loss also affects EMT, considered a crucial step in the early stage of cancer metastasis [19], [20]. However, signal pathways through which FHIT loss might contribute to the metastatic process are not well characterized. In this study, we demonstrate that elevated expression of FHIT leads to reduced motility and invasiveness of lung cancer cells *in vitro* and their ability to metastasize *in vivo*. Concomitantly, FHIT functions as an important modulator of EMT, mediated by transcriptional repression of EMT-related genes. The results indicate that these effects of FHIT could in part be mediated by FHIT-regulated miRNAs, pointing to miRNAs as crucial factors in the metastatic process, and as possible therapeutic targets in cancer.

## Results

### FHIT is a negative regulator of metastasis in lung cancer

**Figure 1:**
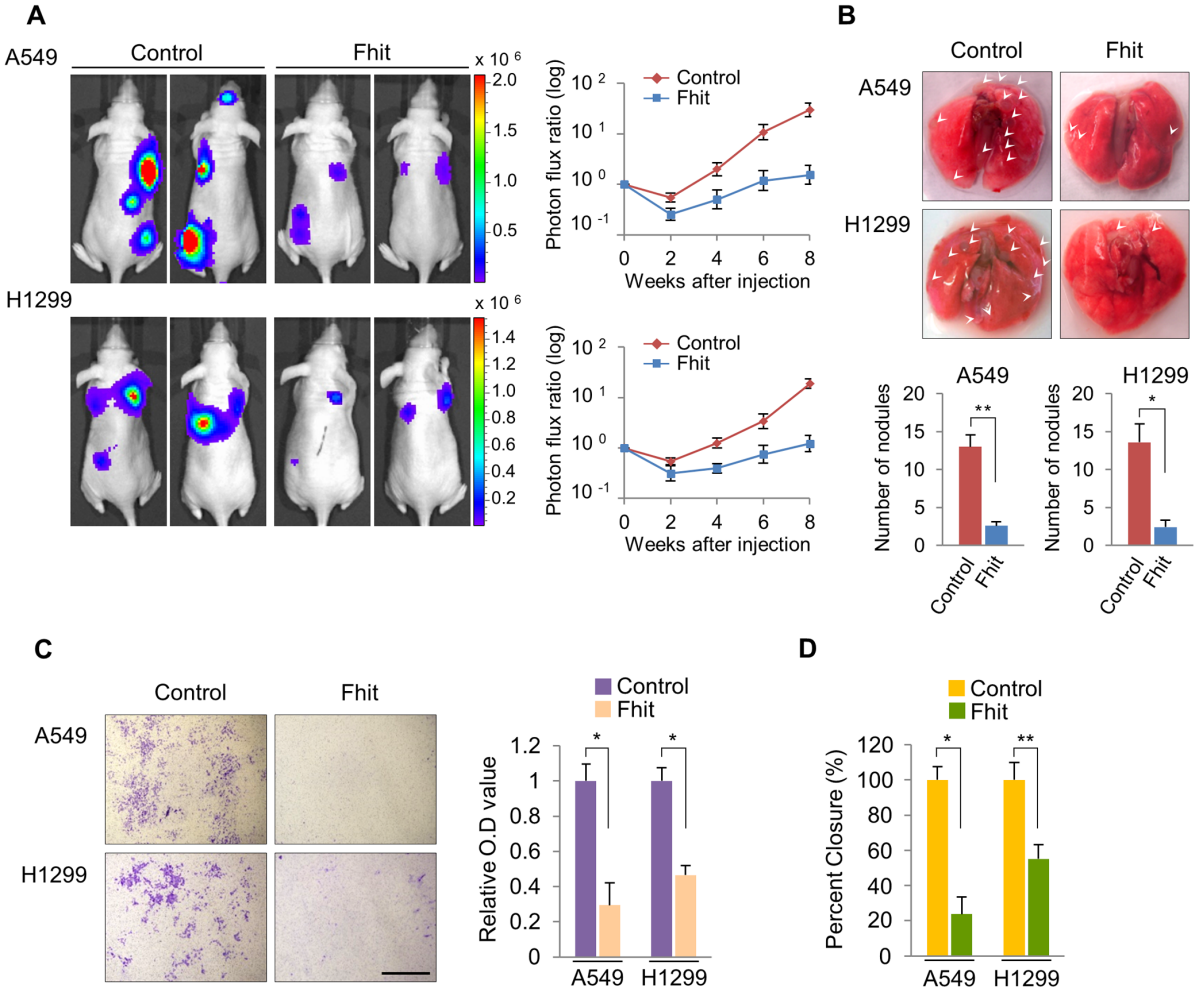
Elevated FHIT expression inhibits metastasis in human NSCLCs. (A) Representative BLI plots of lung metastases in mice after injecting 1×10^6^ A549 or H1299 NSCLC cells stably expressing FHIT or a control vector; n = 4. (B) Lungs were extracted at 8 weeks after tail vein injection and the number of nodules was counted. (C) Representative images of FHIT-expressing or control cells that invaded through the filter and were stained with crystal violet. Scale bar, 40 µm. The results are means ± s.d, n = 4 experiments. *P<0.001. (D) Wound healing assay of confluent monolayer of FHIT-expressing or control A549 and H1299 cells at 0 h and 24 h. See Supplementary Fig. S3, that shows representative images of migration. *P<0.001, **P<0.0001.

The FHIT protein is thought to have a role in tumor suppression based on its expression and function in human cancers as well as its role in tumor development in the FHIT-deficient mouse model. To determine if FHIT could be relevant to the pathogenesis of human lung metastasis, we examined the expression pattern of FHIT in non-metastatic and metastatic lung tissues. Immunostaining for FHIT in lung cancer tissue revealed that FHIT expression was markedly reduced in metastatic tissue compared to non-metastatic (Figure S1, *P* = 0.03). To further investigate the role of FHIT in the metastatic progression of lung cancer, we generated luciferase-tagged human A549 and H1299 non-small-cell lung cancer (NSCLC) cell lines with enforced stable expression of FHIT, and confirmed overexpression of protein by immunoblotting (Figure S2). H1299 and A549 cells show undetectable and moderate levels of endogenous FHIT expression, respectively (Figure S2). Both FHIT expressing cell lines were injected into the arterial circulation of immunodeficient mice by tail vein injection and metastatic capacity evaluated by bioluminescent imaging (BLI). We found that FHIT over-expressing A549 and H1299 cells exhibited reduced metastases in the lung, brain and bone, implying that FHIT may be negatively affecting the extravasation of lung tumor cells (Figure 1A). Continued BLI monitoring revealed a further reduction of metastatic outgrowth in the lungs of animals injected with FHIT expressing cells (Figure 1A). Furthermore, histological analysis confirmed the decrease in the number of metastatic lesions produced by the A549/FHIT and H1299/FHIT cells *vs* control cells (Figure 1B). To analyze metastatic invasion *in vitro*, we measured the infiltration of these cells through Matrigel in a modified Boyden chamber assay and found that the overexpression of FHIT significantly inhibited the invasive capacity (Figure 1C). In addition, the migration activity of both cell lines *via* a wound-healing assay was markedly lower *vs* that of control cells (Figure 1D, S3). Taken together, these results show that FHIT inhibits metastasis of human lung cancer cell lines.

### FHIT inhibits EMT through transcriptional repression of EMT-related genes

**Figure 2:**
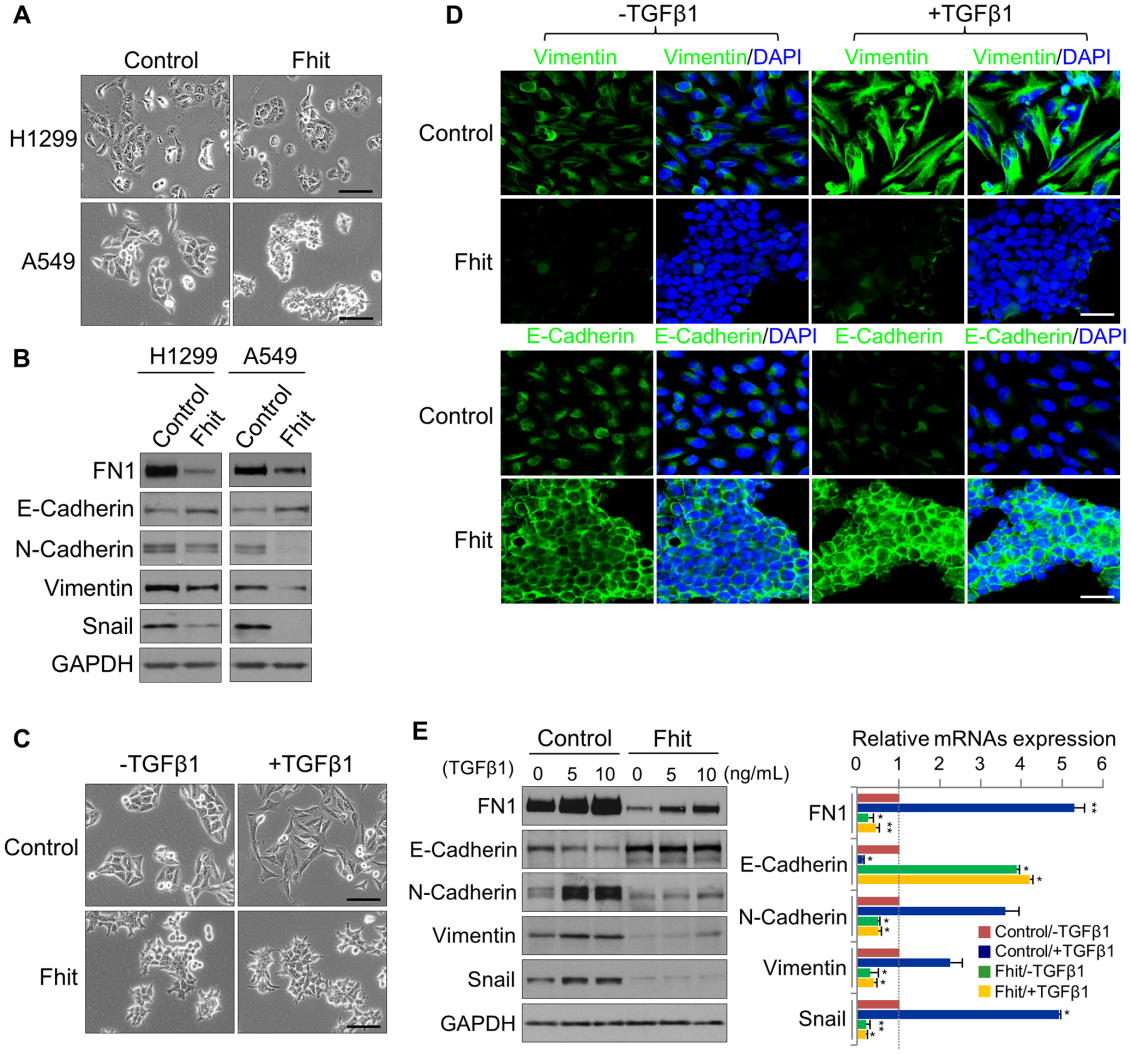
FHIT modulates EMT in NSCLCs through the suppression of EMT-related genes. (A), (B) Phase contrast images of FHIT-expressing or control A549 and H1299 cells, and Immunoblot analysis of EMT markers in both cell lines, respectively. Scale bar, 50 µm. (C), (D) Phase contrast and immunofluorescence images for Vimentin and E-cadherin staining in TGF-β1-induced A549 cells. DAPI staining was used to detect nuclei and merged with Vimentin and E-cadherin stained images. Scale bar, 50 µm (for brightfield images), 20 µm (for immunofluorescence images). (E) Immunoblot and qRT-PCR analyses of expression of EMT markers, Fibronectin (FN1), E-cadherin, N-cadherin, Vimentin and Snail, in response to TGF-β1 treatment of A549 cells with and without FHIT expression. RT-qPCR values were normalized to the housekeeping gene *GAPDH*. Experiments were performed three times and the data are presented as the mean ± s.d. *P<0.05 and **P<0.01 by Student's *t*-test.

It has been reported that FHIT might regulate the expression of genes associated with EMT through modulation of mesenchymal markers, including Vimentin [19]. To assess the mechanism by which FHIT may modulate EMT, we first determined if the epithelial-like characteristics were affected by stable FHIT expression in the lung epithelial cell lines, A459 and H1299. We observed that over-expression of FHIT led to more epithelial properties including increased cuboidal and clustered appearance in both cell lines (Figure 2A). In agreement with the change in cellular appearance, the expression level of the epithelial marker, E-cadherin, was increased, while that of mesenchymal markers, such as Vimentin and Snail, was significantly decreased in both FHIT-expressing cell lines (Figure 2B), suggesting a role for FHIT in enforcing epithelial cellular characteristics. TGF-β signaling is known to play an important role in EMT in various epithelial cells [21], [22]. To examine the effect of FHIT expression on TGF-β-induced EMT, control lung epithelial and A549/FHIT cells were treated with TGF-β and cell morphology was monitored. The FHIT-expressing cells were less or non-responsive to TGF-β1 treatment when compared to control cells showing TGF-β-induced EMT; the typical epithelial phenotype with polygonal morphology and tight arrangement was converted into a spindle-like mesenchymal morphology in control cells, but morphological changes in FHIT-expressing cells were not detected in response to TGF-β1 treatment (Figure 2C). Expression levels of the mesenchymal cell markers Vimentin, Fibronectin, Snail and N-cadherin were suppressed in FHIT-expressing cells, while E-cadherin levels were elevated (Figure 2D, 2E, S4). Expression levels of mesenchymal markers were constantly suppressed in A549/FHIT cells at translational and transcriptional levels, whereas control cells showed elevation in response to TGF-β1 (Figure 2D, 2E, S4). In addition, down-regulation of E-cadherin by TGF-β1 was not observed in FHIT-expressing A549 cells (Figure 2D, 2E). These data suggest that FHIT inhibits EMT by repressing or activating EMT-related genes and contributing to insensitivity to TGF-β-induced EMT.

### A FHIT*-*activated miRNA, miR-30c, inhibits EMT through suppression of direct targets, Vimentin and Fibronectin

**Figure 3:**
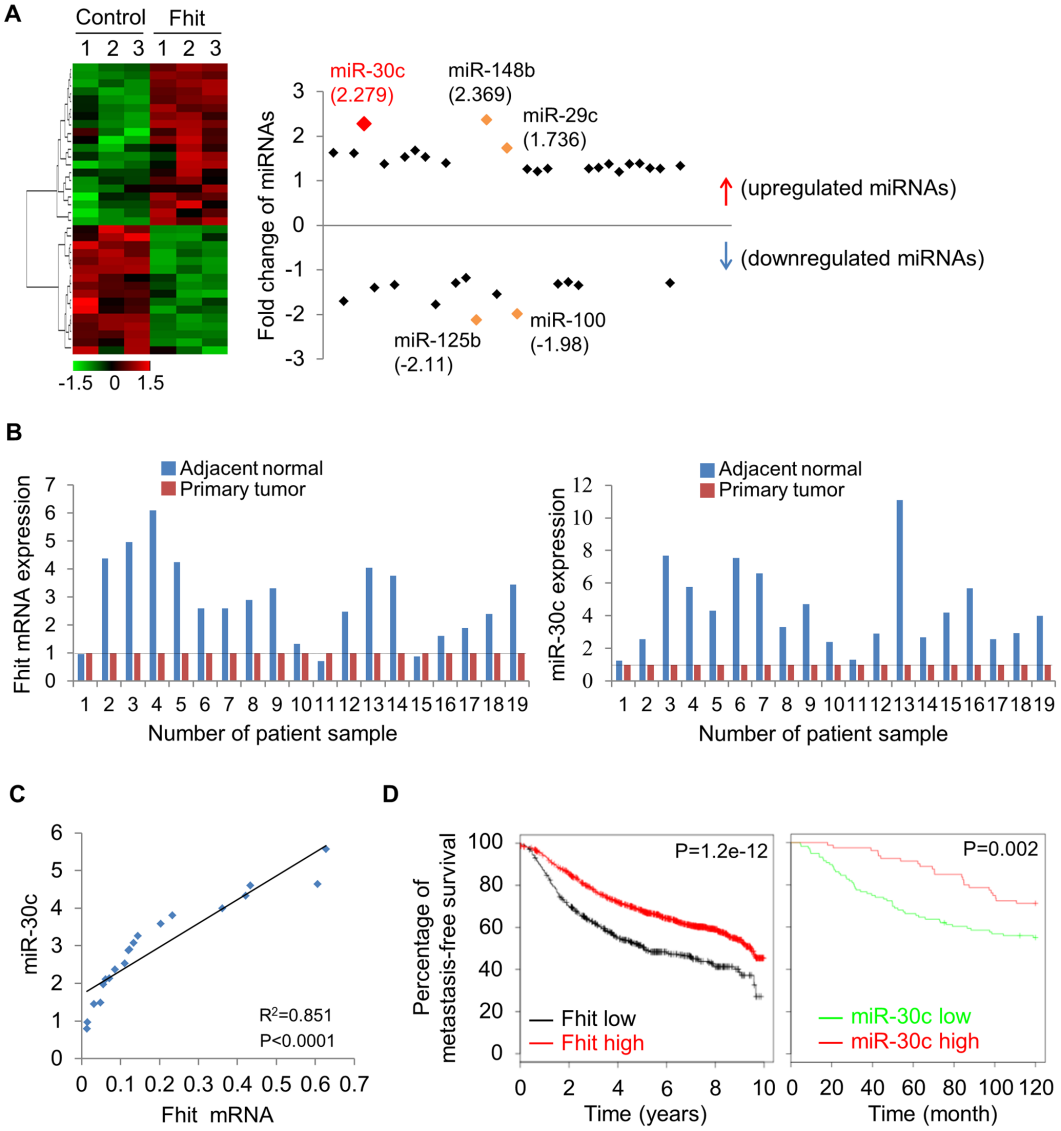
FHIT expression leads to activation of miR-30c in lung cancer. (A) Unsupervised hierarchical clustering of FHIT-regulated miRNAs in H1299/FHIT cells; *P*<0.05 (B) RT-qPCR analysis showing up-regulation of FHIT and miR-30c in adjacent normal tissues compared to tumor tissues (n = 19). (C) XY scatter plots showing positive correlation between FHIT and miR-30c in primary lung tumors (n = 20). (D) Kaplan-Meier plots of metastasis-free survival of breast cancer patients, stratified by expression of FHIT (2290 patients) or miR-30c (210 patients). Data obtained from the Kaplan-Meier plotter database (kmplot.com/analysis) or microRNA survival analyses in cancer (www.bioprofiling.de).

Due to the critical involvement of microRNAs (miRNAs) in cancer metastasis and EMT, we next determined if FHIT might regulate expression of specific miRNAs. To identify miRNAs regulated by FHIT, we used the nanoString nCounter platform for profiling expression of miRNAs in the H1299/FHIT and control cells; miRNAs up-regulated by >1.2 fold change and an expression level higher than 100 code counts were further analyzed (Figure 3A, Table S1). Among the miRNAs active in H1299/FHIT cells, the second most up-regulated miRNA, miR-30c, stood out as an attractive candidate for a role in FHIT-related function (median fold change: 2.279, *P*<0.0001). To validate the profile data, we performed stem-loop real-time quantitative PCR analysis for miR-30c, using RNAs from H1299/FHIT cells and A549/FHIT. We confirmed that miR-30c expression was higher in both FHIT-expressing cells *vs* control cells (Figure S5). To explore the *in vivo* expression pattern of FHIT and miR-30c, total RNAs from human primary lung tumors and adjacent normal tissues (n = 19) were extracted, and RT-qPCR analyses performed. We observed that FHIT (17/19 cases) and miR-30c (19/19 cases) expression levels were positively correlated in adjacent normal tissues and up-regulated as compared to primary lung tissues (Figure 3B, S6). Also, the expression of FHIT and miR-30c was positively correlated in primary lung cancer tumors (n = 20, coefficient  = 0.851 and *P*<0.0001) (Figure 3C). We further evaluated the prognostic value of FHIT and miR-30c in a large public clinical microarray database [23], [24] and found trends towards improved metastasis-free survival on breast cancers in cases of high expression of FHIT and miR-30c (Figure 3D), suggesting that their associated expression may function to oppose cancer progression.

**Figure 4:**
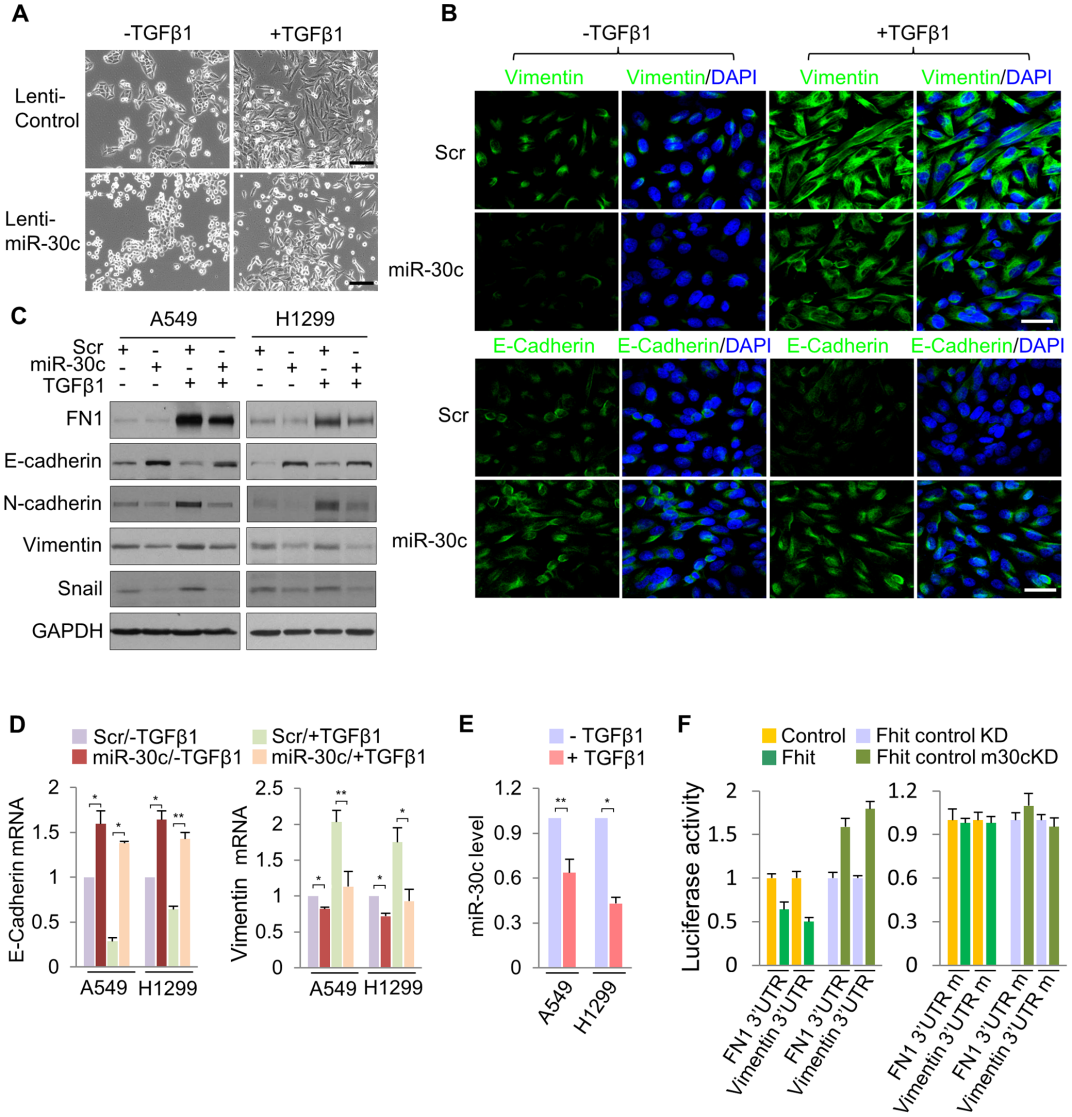
miR-30c inhibits TGF-β-induced EMT through direct targeting of Vimentin and Fibronectin. (A) Phase contrast images of A549/miR-30c cells in response to TGF-β1 treatment. Scale bar, 100 µm. (B), (C) Immunofluorescence images and Western blot analysis of EMT markers, E-cadherin, FN1 and Vimentin, in miR-30c-transfected A549 cells treated with TGF-β1, respectively. Scale bar, 20 µm. (D) RT-qPCR analysis showing induction of E-cadherin and a decline in the expression of Vimentin in TGF-β-induced A549/miR-30c cells. *P<0.05 and **P<0.01 by Student's *t*-test. (E) RT-qPCR for miR-30c in response to TGF-β1 treatment. *P<0.001 by Student's *t*-test. (F) miR-30c down-regulates mesenchymal markers, Vimentin and Fibronectin by directly targeting their 3′ UTRs. Luciferase reporters assays using wild type or mutant 3′ UTR were performed after transfection into A549/FHIT or A549/FHIT cells with miR-30c knockdown. Experiments were performed three times and the data are presented as the mean ± s.d.

To determine if miR-30c is implicated in EMT, we investigated its role upon expression in A549 cells induced to undergo EMT in response to TGF-β1 treatment. We first examined the morphology of A549 cells cultivated in the presence of TGF-β treatment. As shown in Figure 4A, A549/miR-30c cells exhibited reduction of EMT-like morphological features *vs* control cells which were converted from a predominant epithelial phenotype to an EMT phenotype within 48 hr in response to TGF-β1 treatment (Figure 4A). Consistently, at the molecular level, miR-30c expression led to decreased levels of Fibronectin and Vimentin and increased E-cadherin levels (Figure 4B, 4C). Furthermore, TGF-β-induced expression of the mesenchymal markers, Fibronectin and Vimentin, was suppressed in the presence of miR-30c, while TGF-β-repressed E-cadherin was markedly activated (Figure 4C), followed by transcriptional activation and suppression of E-cadherin and Vimentin, respectively (Figure 4D). Interestingly, we observed significant reduction of miR-30c levels in TGF-β treated A549 and H1299 cell lines (Figure 4E). Next, we set out to identify putative target genes of miR-30c, which could mediate the inhibition of EMT induced by TGF-β, by using miRNA-target-predicting software PicTar and TargetScan 5.1 or RNA22 program. We found miR-30c binding sites in the 3′UTRs of Vimentin and Fibronectin genes (Figure S7), supporting their candidacy as miR-30c targets. To verify that they are direct targets of miR-30c, their 3'UTRs containing miRNA-responsive elements were cloned into the pGL3 construct downstream of the luciferase ORF. The reporter activity was markedly suppressed by the presence of the 3'UTRs of Vimentin and Fibronectin in A549/Fhit cells compared to the control cells, which reversed when the 3'UTR was mutated, whereas it was increased in A549/Fhit cells with knockdown of miR-30c (Figure 4F). In addition, we found that, in the presence of miR-30c, Vimentin and Fibronectin protein levels decreased in H1299 and A549 cells (Figure S7). In addition, we observed that Vimentin mRNA, but not Fibronectin mRNA, was decreased in miR-30c-transfected lung cancer cells, A549 and H1299 (Figure S7). Interestingly, the expression of Vimentin mRNA was inversely correlated with that of miR-30c in the adjacent normal tissues and matched tumors (Figure S8). Taken together, the results strongly suggest that miR-30c can regulate EMT through down-regulation of the mesenchymal markers, Fibronectin and Vimentin, by directly targeting their 3′UTRs.

### miR-30c targets metastasis-related genes, *MTDH* and *HMGA2*

**Figure 5:**
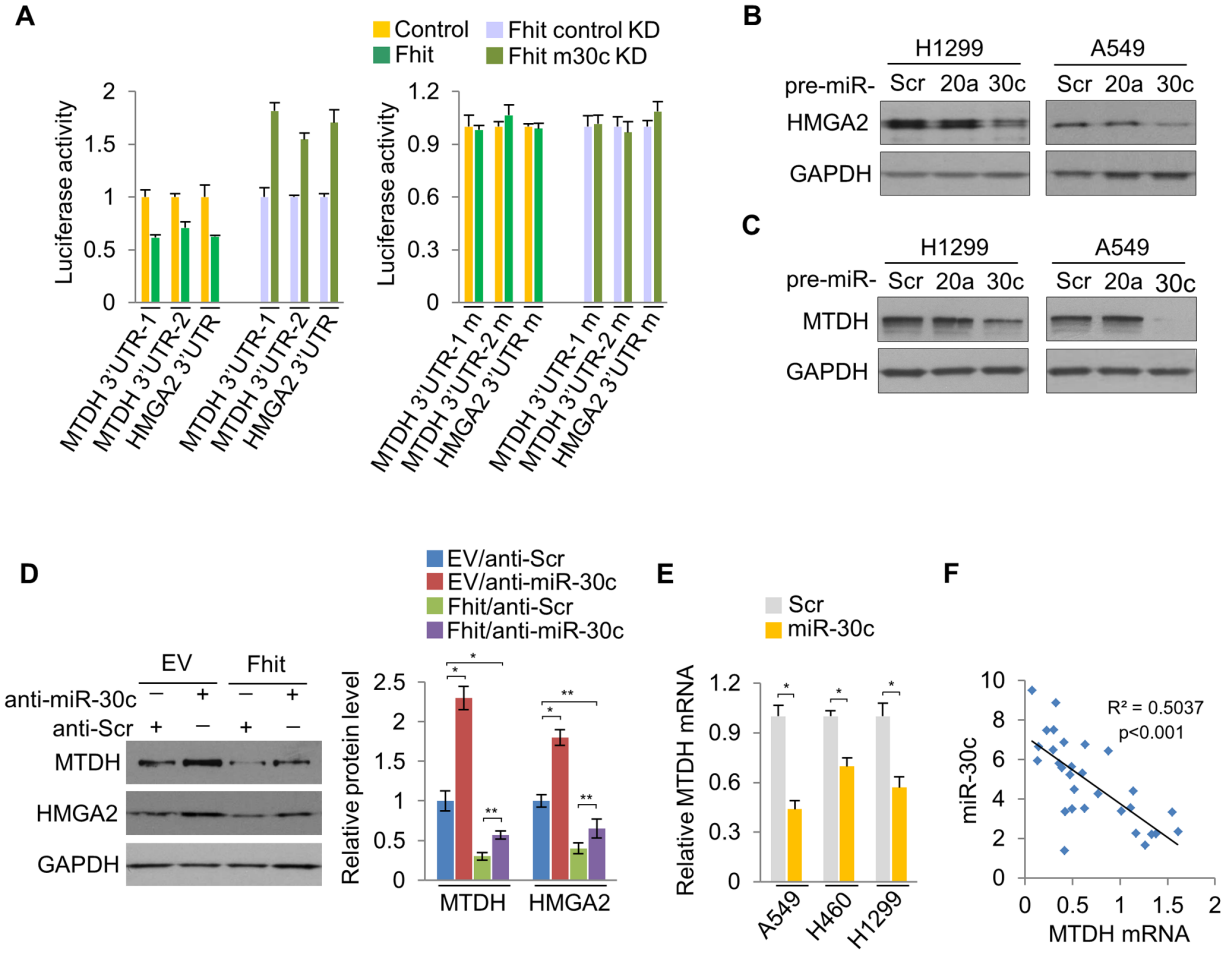
miR-30c down-regulates the expression of MTDH and HMGA2 by directly targeting their 3′UTRs. (A) Luciferase activity assays of luciferase reporters with wild type or mutant 3′UTRs of MTDH or HMGA2 were performed after transfection into A549/FHIT or A549/FHIT cells with miR-30c knockdown. (B), (C) Western blot analysis for HMGA2 or MTDH in miR-30c-transfected cells, H1299 and A549. miR-20a was another negative control. (D) MTDH or HMGA2 protein levels in anti-miR-30c-transfected cells stably expressing FHIT. (E) RT-qPCR for MTDH in presence of miR-30c. (F) Correlation between expression levels of miR-30c and MTDH in NSCLC samples (n = 30). Pearson's correlation was used. y, the relative expression level of miR-30c, U6, as an internal control. x, the relative expression level of MTDH, GAPDH, as an internal control. Experiments were performed three times and the data are presented as the mean ± s.d. *P<0.01 and **P<0.001 by Student's *t*-test.

Next, we determined if miR-30c targets other genes implicated in metastasis, bridging the connections among miR-30c, metastasis and EMT. Bioinformatics analyses indicated that miR-30c might target several other key mRNAs, including metastasis-associated genes metadherin (*MTDH*) and High-mobility group AT—hook 2 (*HMGA2*). We found that there are four binding sites for miR-30c in *MTDH* 3′UTR and one binding site in *HMGA2* (Figure S9). These two genes, *MTDH* and *HMGA2*, are known as oncogenes in a variety of human cancers; MTDH protein is over-expressed and promotes metastatic seeding in breast cancer and over-expression of HMGA2 also induces metastasis and invasion of human cancer cells [25], [26]. In addition, the levels of MTDH and HMGA2 mRNAs were highly up-regulated in primary tumors compared to their matched adjacent normal tissues (Figure S10). To confirm that MTDH and HMGA2 are direct targets of miR-30c, luciferase assays were performed. Luciferase activity was suppressed by the wild type 3'UTRs of these genes in A549/Fhit cells compared to the control cells, which was reversed when the 3'UTR was mutated, whereas it was increased in A549/Fhit cells with knockdown of miR-30c (Figure 5A). In immunoblot assays (Figure 5B, 5C), ectopic expression of miR-30c led to significantly decreased levels of MTDH and HMGA2 in H1299 and A549 cells. Furthermore, the expression of MTDH and HMGA2 in FHIT-expressing H1299 cells, in which miR-30c is up-regulated, was severely suppressed and miR-30c silencing resulted in the increase in their expression levels (Figure 5D). However, miR-30c silencing was insufficient to fully rescue this phenotype (Figure 5D), suggesting that additional mechanisms contribute to FHIT regulation of MTDH and HMGA2. In addition, we observed that MTDH mRNA, but not HMGA2 mRNA, was decreased in miR-30c-transfected lung cancer cells, A549, H460 and H1299 (Figure 5E) and showed inverse correlation with miR-30c in lung primary tumors (Figure 5F). Taken together, these data suggested that *MTDH* and *HMGA2* are direct target genes for miR-30c in lung cancer.

### miR-30c suppresses metastasis through the suppression of MTDH and HMGA2

**Figure 6:**
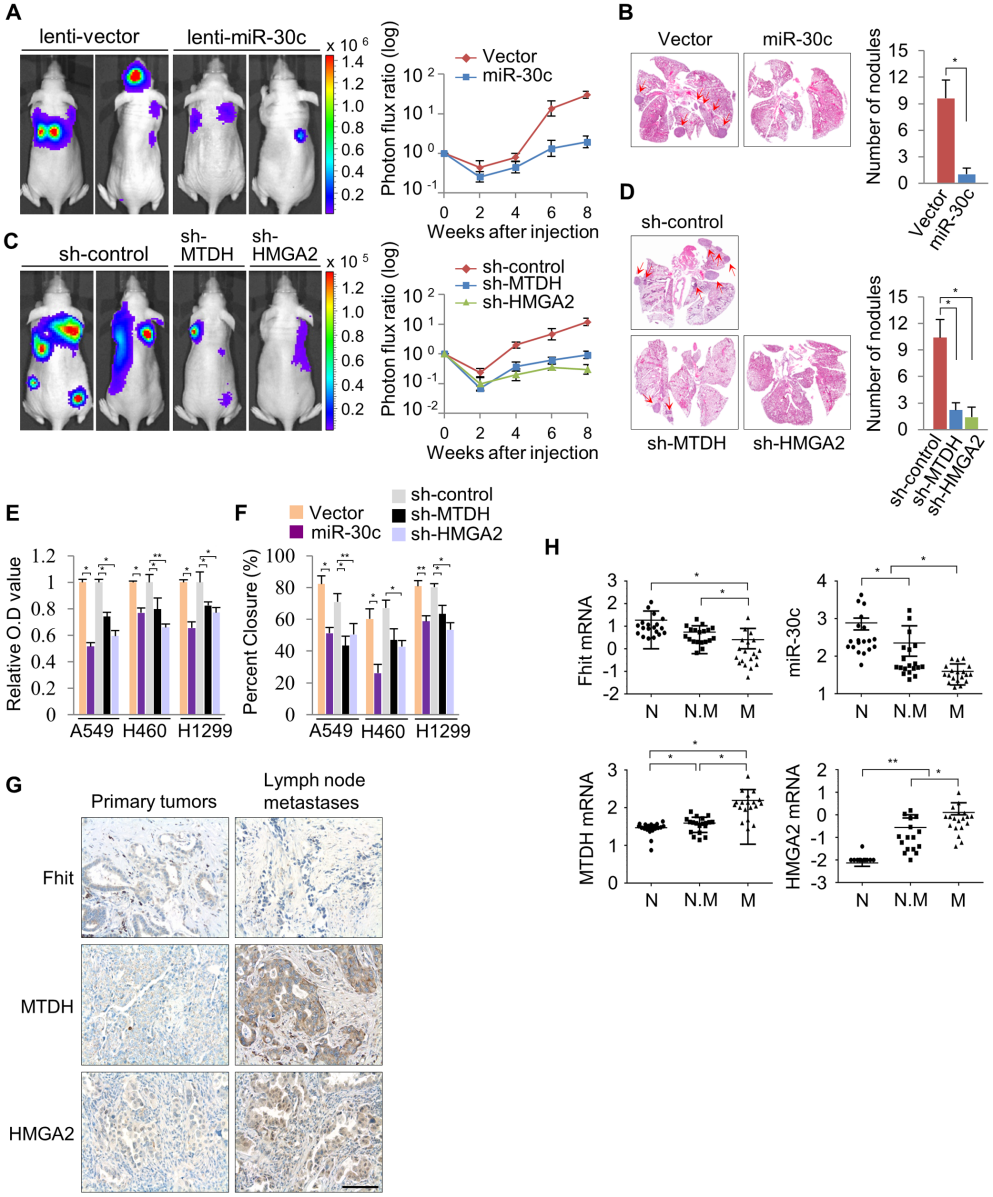
miR-30c inhibits metastasis through the suppression of MTDH and HMGA2 in NSCLCs. (A) Representative BLI plots of lung metastasis of mice injected (IV) with 1×10^6^ A549 cells stably expressing miR-30c or a control vector; n = 4. (B) Representative H&E stained lung section. The arrows highlight metastatic nodules. *P<0.05 by Student's *t*-test. (C) Representative BLI plot of metastasis after IV injection with 1×10^6^ A549 cells after knockdown of MTDH, HMGA2, or control; n = 5. (D) Representative H&E stained lung section and the number of lung metastatic nodules. The arrows indicate metastatic nodules. *P<0.001. (E), (F) Invasion and migration assays in control and miR-30c-overexpressing cells, or control, MTDH and HMGA2 knockdown cells, respectively. *P<0.001 **P<0.01 by Student's *t*-test. (G) Immunohistochemistry assay for FHIT, MTDH and HMGA2 in primary lung tumors and their matched lymph node metastases. Scale bar, 100 µm. (H) The expression pattern of FHIT, miR-30c, MTDH, and HMGA2 in normal (N), non-metastatic (N.M) and metastatic tissues (M). *P<0.001 **P<0.05 by Student's *t*-test.

To determine if miR-30c is capable of suppressing metastasis in lung cancer, this miR was stably introduced into A549 cells. These cells were injected into immunodeficient mice and evaluated for metastatic colonization capacity in a tail-vein assay. We found, *via* BLI monitoring, that the over-expression of miR-30c in parental A549 lung cancer cells potently suppressed pulmonary metastases in the lung and brain (Figure 6A). Histological quantification revealed that there is a significant reduction in the total number of miR-30c expressing metastatic nodules in lungs (Figure 6B). To further clarify the mechanism whereby miR-30c suppresses metastasis, we generated stable knockdown cells for its target genes, *MTDH* or *HMGA2*, by using independent short hairpins and confirmed knockdown of protein by immunoblotting (Figure S11). These MTDH and HMGA2 depleted cells were inoculated into *nude* mice *via* tail vein. The knockdown of these genes led to significant suppression of metastatic colonization in the lung and bone (Figure 6C). By histological quantification, a significant reduction in the total number of metastatic nodules in knockdown cells was observed *vs* control cells (Figure 6D). In accord with these results, lung cancer cells, A549, H460 and H1299, with enhanced expression of miR-30c, or MTDH or HMGA2 knockdown exhibited a decrease in cell invasion and migration *in vitro* (Figure 6E, 6F). Finally, to determine if our experimental findings could be relevant to the pathogenesis of human lung cancer, we examined the expression pattern of FHIT, miR-30c, MTDH and HMGA2 in primary tumor tissues and matched lymph node metastatic tissues. Immunohistochemical staining showed that FHIT expression was decreased in matched lymph node metastatic tissues compared to primary tumor tissue (9 of 40 cases), but MTDH and HMGA2 were significantly activated (11 of 40 and 10/40 cases, respectively) in the matched lymph nodes (Figure 6G). Using RT-qPCR analysis, we found that FHIT mRNA and miR-30c expression levels were inversely correlated with expression levels of MTDH and HMGA2: FHIT and miR-30c levels were more highly expressed in the primary tumors relative to expression in the matched lymph node tissues, while MTDH and HMGA2 mRNAs were increased in matched lymph node tissues (Figure S12). Next, we were interested in identifying the expression patterns of FHIT, miR-30c, MTDH and HMGA2 in lung cancer progression and examined their mRNA levels in normal, non-metastatic and metastatic lung tissues. Interestingly, we found that FHIT and miR-30c gradually decreased with metastasis progression, while MTDH and HMGA2 expression increased (Figure 6H). Overall, these data strongly suggest that FHIT and miR-30c inhibit metastasis through targeting the metastasis-related genes, MTDH and HMGA2.

## Discussion

**Figure 7:**
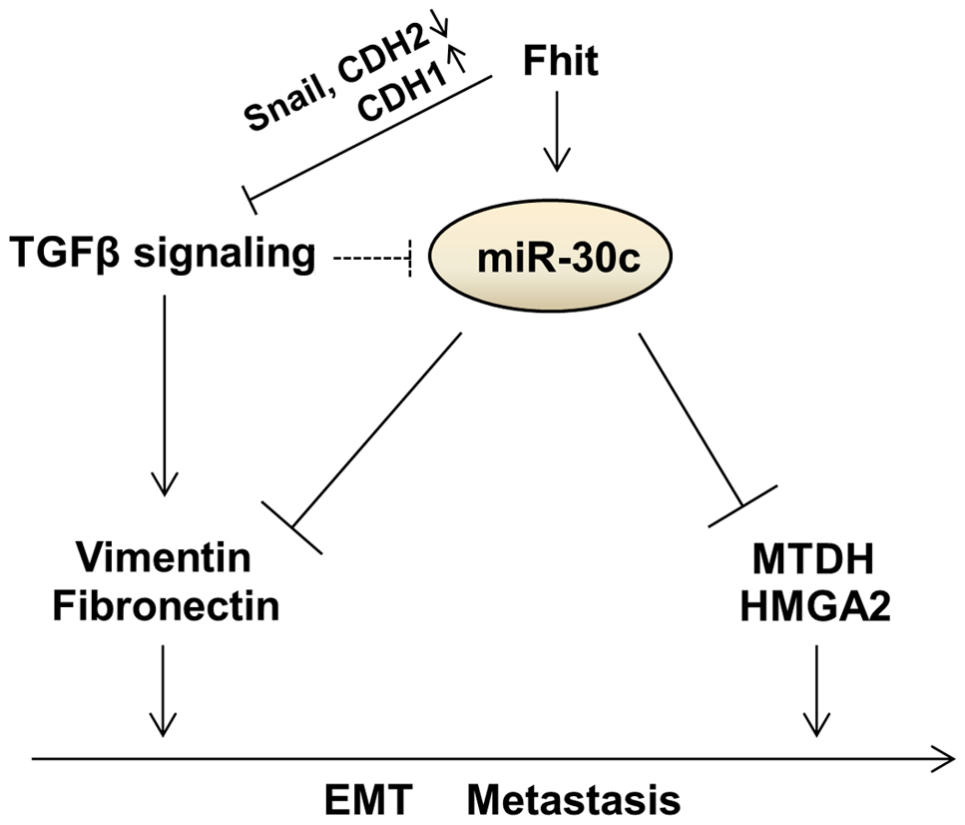
Proposed model for the function of the FHIT and miR-30c in lung metastasis and EMT.

The *FHIT* gene has been implicated extensively in human tumor progression and plays multiple biological roles in inhibiting tumorigenesis [27], [28]. Although some studies showed the trends of reduced FHIT expression in microinvasive and invasive carcinoma, and a relationship between FHIT expression and tumor invasion, EMT and metastasis [19], [20], [29], [30], our study revealed an unrecognized role for FHIT in modulation of expression of miRNA genes. Using *in vivo* and *in vitro* models, we show the ability of FHIT to inhibit metastasis and EMT through the suppression of metastasis-related and EMT-associated genes including *MTDH*, *HMGA2*, *VIM*, *FN1*, *CDH1* and *Snail* in non-small-cell lung cancer (NSCLC) cells. In particular, FHIT severely suppresses TGF-β-induced EMT, suppressing changes in cellular morphology and levels of EMT-related markers, implying that FHIT serves as a master enforcer of the epithelial cell fate. Here, we identified novel miRNAs modulated by FHIT in NSCLC and delineated the role of one FHIT-regulated miRNA, miR-30c, in regulating EMT and metastasis. Our results show that miR-30c functions as a negative regulator of EMT and metastasis through directly targeting mesenchymal markers, Vimentin and Fibronectin, and metastasis-related genes, MTDH and HMGA2, implying that miR-30c contributes to the FHIT regulation of EMT and metastasis (Figure 7). In fact, this miRNA may function as an anti-metastatic miRNA in several types of human cancer; it has been shown that miR-30 inhibits metastasis *in vivo* and *in vitro* in a metastatic breast cancer model [31], [32]. In hepatocellular carcinomas, miR-30c was identified as a metastasis-related miRNA, which is down-regulated in metastatic relative to non-metastatic lesions and is associated with improved survival [33]. Our findings further confirm miR-30c anti-metastatic function *via* targeting MTDH and HMGA2 and correlation with improved prognosis. In addition, in agreement with previous studies showing that the miR-30 family members inhibit the EMT process and confer epithelial phenotype to cancer cells including pancreatic and hepatocellular carcinomas [34], [35], our data demonstrated that FHIT-activated miR-30c inhibits TGF-β-induced EMT in NSCLC A549 cells through direct targeting of mesenchymal markers, VIM and FN1, and activation of epithelial marker and metastasis suppressor, E-cadherin (Figure 6I). Interestingly, the expression level of miR-30c was significantly down-regulated in TGF-β-treated A549 and H460 cells, suggesting that there is a reciprocal regulatory relationship between TGF-β signaling and miR-30c (Figure 6I). Notably, patients whose primary tumors were positive for FHIT and miR-30c have a significantly improved metastasis-free survival, implying that FHIT and miR-30c might be better used as a predictor of overall metastasis rather than lung- or bone-specific metastasis. However, knockdown of miR-30c in FHIT stable A549 cells could not overcome FHIT-suppressed metastasis *in vivo*. Furthermore, miR-30c cannot efficiently inhibit TGF-β-induced EMT in respect of morphological and molecular changes as FHIT does. Since this role of miR-30c seems to be necessary but not sufficient for mediating FHIT-dependent suppression of metastasis and EMT suppression, other FHIT-regulated targets must also operate to enable metastasis and EMT suppression. In addition, expression levels of miR-30c were not dramatically induced in FHIT overexpressing cells, being ∼2-fold higher than in the control. These facts suggest that the effect of FHIT on suppression of metastasis is in part dependent on the function of FHIT-activated miR-30c, showing that miR-30c functions as one mediator of FHIT-modulated metastasis, but not the only mediator. Interestingly, FHIT loss has been detected frequently during the early onset of disease progression in cancer [16] and the expression of FHIT and miR-30c is gradually decreased during tumor progression (normal tissues < non-metastatic tumors < metastatic tumors), while expression of miR-30c target genes, MTDH and HMGA2, is increased (Figure 6H). These observations suggest that miR-30c expression is often reduced at early stages of tumor progression when decreased FHIT expression is already apparent and therefore represents one of the driving forces for early stage lung tumor cells to proceed with EMT and subsequent metastatic progression, thus highlighting the relationship between FHIT and miR-30c as potential targets for early therapeutic intervention in lung cancer progression.

## Materials and Methods

### Ethics statement

All animals were housed in the Ohio State University animal facility and the experiments with live animals were approved by our institute animal committee (IACUC 2013-A0146) and conducted following the Ohio State University animal policy in accordance with NIH guidelines. Every effort was made to minimize the animals' suffering, anxiety, and discomfort.

### Metastasis assays in nude mice

1×10^6^ cells were washed in PBS and injected intravenously into four- to six-week-old age- matched female NOD/SCID mice to study lung metastasis activity as described previously [36]. Noninvasive bioluminescence imaging was performed every two weeks after injection to quantify the metastasis burden using an IVIS 200 Imaging System (Caliper Life Sciences) as described previously [36].

### Lung cancer samples and cell lines

192 and 80 lung cancer tissues were purchased from US Biomax, Inc. 40 lung tumor tissue samples were provided from the Department of Pathology, Ohio State University. All human tissues were obtained according to a protocol approved by the Ohio State Institutional Review Board. A549, H460, H1299 cell lines were grown in RPMI containing 10% heat-inactivated FBS and with 2 mM L-glutamine and 100 Uml^-1^ penicillin–streptomycin.

### Invasion assay

Transwell insert chambers with an 8- µm porous membrane (Bio-Rad) were used for the Invasion assay. Cells were washed three times with PBS and added to the top chamber in serum-free medium. The bottom chamber was filled with medium containing 10% FBS. Cells were incubated for 48 hrs at 37°C in a 5% CO_2_ humidified incubator. To quantify invaded cells, cells in the top chamber were removed by using a cotton-tipped swab, and the invaded cells were fixed in PBS, 25% glutaraldehyde and stained with crystal violet stain, visualized under a phase-contrast microscope and photographed. Crystal-violet-stained cells were then solubilized in acetic acid and methanol (1∶1), and absorbance was measured at 595 nm.

### Scratch assay

Fhit-overexpressing A549 and H1299 or A549, H460 and H1299 stably expressing miR-30c were incubated with medium 5% FBS. Images were acquired directly after scratching (0 h) and after 24 h. Quantization of migration distance using Image J software. The distance covered was calculated by converting pixel to millimeters.

### shRNA lentiviral particles transduction

Cells were plated in a 12-well plate 24 hours prior to viral infection and incubated overnight with 1 ml of complete optimal medium (with serum and antibiotics). The day after the medium was removed and 1 ml of complete medium with Polybrene (5 µg/ml) was added. The day after, cells were infected by adding 50 µl of control shRNA, shMTDH, shHMGA2 Lentiviral Particles (Santa Cruz) to the cultures. Stable clones were selected via 1 µg/ml of Puromycin dihydrochloride.

### Immunofluorescence

Cells were grown on Lab-Tek II CC2 chamber slides (Nunc), fixed with 4% paraformaldehyde and permeabilized with 0.2% Triton X-100/PBS before blocking with 10% sheep serum (Caltag Laboratories). All the primary antibodies were from Abcam. Secondary antibodies were goat antibodies to mouse or rabbit coupled to Alexa 488 (Invitrogen). Cell nuclei were visualized with DAPI (Sigma). Slides were mounted with SlowFade Gold Antifade reagent (Invitrogen).

### Generation of stable clones

A549 cells were stably infected with the Human pre-microRNA Expression Construct Lenti-miR expression plasmid containing the full-length *miR-30c* and the GFP gene under the control of two different promoters (System Biosciences), or the pGreenPuro shRNA expression lentivector containing the miRZip short hairpin of anti-sense *miR-30c* (System Biosciences). For lentiviral vectors expressing the Fhit protein, a 707 bp fragment of *FHIT* cDNA was amplified by reverse transcriptional PCR from human placental cDNA and cloned into a lentiviral shuttle vector (pCDH-CMV-MCS-EF1-RFP) purchased from System Biosciences (CA, USA). An empty vector was used as control. They were packaged with pPACKH1 Lentivector Packaging Plasmid mix (System Biosciences) in a 293TN packaging cell line. Viruses were concentrated using PEGit Virus Precipitation Solution, and titers were analyzed using the UltraRapid Lentiviral Titer Kit (System Biosciences). Infected cells were selected by FACS analysis (FACScalibur; BD Bioscience). Infection efficiency >90% was verified by fluorescent microscopy and confirmed by for miRs expression.

### NanoString nCounter assay

The NanoString nCounter Human miRNA expression Assay Kit (http://www.nanostring.com) was used to profile more than 700 human and human-viral miRNAs in U87 cells treated with Nutlin-3a (10 µM) and DMSO. 100 ng of total RNA was used as input for nCounter miRNA sample preparation reactions. All sample preparation was performed according to manufacturer's instructions (NanoString Technologies). Preparation of small RNA samples involves the ligation of a specific DNA tag onto the 3′ end of each mature miRNA. These tags are designed to normalize the Tm's of the miRNAs as well as to provide a unique identification for each miRNA species in the samples. The tagging is accomplished in a multiplexed ligation reaction using reverse-complementary bridge oligonucleotides to direct the ligation of each miRNA to its designated tag. Following the ligation reaction, excess tags and bridges are removed, and the resulting material is hybridized with a panel of miRNA: tag-specific nCounter capture and barcoded reporter probes. Hybridization reactions were performed according to manufacturer's instructions with 5 µl of the 5-fold diluted sample preparation reaction. All hybridization reactions were incubated at 64°C for a minimum of 18 hr. Hybridized probes were purified using the nCounter Prep Station (NanoString Technologies) following the manufacturer's instructions to remove excess capture and reporter probes and to immobilize transcript-specific ternary complexes on a sterptavidin-coated cartridge. Date collection was carried out on the nCounter Digital Analyzer (NanoString Technologies) following the manufacturer's instructions to count individual fluorescent barcodes and quantify target RNA molecules present in each sample. For each assay, a high density (600 fields of view) was performed.

### Western blot analysis

Samples were extracted in 15 mM Tris_Cl, pH 7.5/120 mM NaCl/25 mM KCl/2 mM EGTA/0.1 mM DTT/0.5% Triton X-100/10 mg/ml leupeptin/0.5 mM PMSF. Total protein (50 µg) from each sample was separated on a 4–20% Tris-HCl Criterion precast gel Bio-Rad (cat# 345-0032, Hercules, CA) and transferred to a poly (vinylidene difluoride) filter (Millipore). The filter was blocked in 5% nonfat dry milk, incubated with the specific antibody, washed, and probed with secondary antibody IgG conjugated to horseradish peroxidase (Santa Cruz Biotechnology), and developed with enhanced chemiluminescence (Amersham Pharmacia). Immunoblot analyses were performed using the following antibodies: Fibronectin (Cat#F3648, Sigma-Aldrich), E-cadherin (Cat#5409-1, Epitomics, Inc), Vimentin (Cat#2862-1, Epitomics, Inc), Snail (Cat#sc-28199, Santa Cruz Biotechnology), N-cadherin (Cat#610920, BD Biosciences), HMGA2 (Cat#5269, Cell Signaling), MTDH (Cat#AB2989, Millipore) and GAPDH (Cat#2118, Cell Signaling).

### RNA extraction and RT-PCR

Total RNA was extracted using TRIzol Reagent Invitrogen (Cat# 15596-018) following the manufacture's instruction. Specifically the pellet obtained from 5×10^6^ cells was lysed 1 ml of TRIzol solution. At the end of the extraction the isolated RNA was dissolved in 35 µl in RNase-free water and incubated for 10 min at 55°C. An aliquot of 5 µg RNA was then used for cDNA synthesis using the SuperScript first strand cDNA synthesis kit (Invitrogen). RT-PCRs were carried out using ABI Prism 7900HT sequence detection systems with Applied Biosystems TaqMan Gene expression assays (*miR-30c*: 000419; Fibronectin: Hs00365052_m1; E-cadherin: Hs00154405_m1; N-cadherin: Hs00983056_m1; Vimentin: Hs00185584_m1; Snail: Hs00195591_m1: Fhit: Hs00179987_m1; MTDH: 00757841_m1).

### Immunohistochemistry

Immunohistochemical staining was performed to detect the expression of Fhit in primary lung tissue or Fhit, MTDH and HMGA2 in primary lung tissue and matched lymph node metastasis tissue. The primary antibody against Fhit was provided by Dr. Kay Huebner (The Ohio State University, 1∶100), MTDH and HMGA2 primary antibody was obtained from Epitomics, Inc and Cell signaling, respectively. Scoring was measured by the percentage of positive cells with the following staining intensities: the signal (+) indicates that at least 10% cancer cells with signal required for positive results are present in tissue.

### Luciferase reporter vector

The 3′UTR of the human Vimentin, Fibronectin, *MTDH* and *HMGA2* genes were PCR amplified (Text S1). They were then cloned downstream of the Renilla luciferase stop codon in pGL3 control vector (Promega), giving rise to the p3′UTR-Vimentin, -Fibronectin, -MTDH and –HMGA2 plasmids. These constructs were used to generate, by inverse PCR, the p3′UTRmut-Vimentin, Fibronectin, MTDH and –HMGA2 plasmid (Text S1). Fhitrexpressing A549 cells or *miR-30c* knockdown Fhit-expressing A540 cells were cotransfected with 1 µg of each construct and 0.1 µg of a Renilla luciferase expression construct, pRL-TK (Promega), using Lipofetamine 2000 (Invitrogen). Cells were harvested 24 hr after transfection and assayed with Dual Luciferase Assay (Promega) according to the manufacturer's instructions. Three independent experiments were performed in triplicate.

### Statistical analysis

Student's t test and one-way analysis of variance was used to determine significance. All error bars represent the standard error of the mean. Statistical significance for all the tests, assessed by calculating p value, was <0.05. Spearman correlation coefficient was calculated to test the association between *miR-30c* and Fhit mRNA in lung samples (n = 19). Kruskal-Wallis was used to assess whether the *miR-30c* and Fhit are differentially expressed among normal lung and primary samples on the basis of the Bartlett test P value. The in vivo metastasis-free survival of *miR-30* and Fhit was assessed by plotting survival curves according to the Kaplan–Meier method, and groups were compared using the log-rank test.

## Supporting Information

Figure S1 Immunohistochemistry assay to detect Fhit expression in Non-metastatic and Metastatic tumors.
(TIF)

Figure S2 The expression of Fhit protein in Fhit-overexpressing cells, A549 and H1299 were measured by western blotting.
(TIF)

Figure S3 Representative photographs of scratched areas of the confluent monolayer of A549 (A) or H1299 cells (B) stably expressing Fhit or control vector at 0 h and 24 h after wounding with a pipet tip. Scale bar, 500 µm.
(TIF)

Figure S4 Immunofluorescence assay for N-cadherin in Fhit-expressing or control A549 cells treated with TGF-β-1. Scale bar, 20 µm.
(TIF)

Figure S5 The expression levels of *miR-30c* in *miR-30c*-overexpressing cells, A549 and H1299. * *P*<0.05 by Student's *t*-test.
(TIF)

Figure S6 The different expression levels of Fhit and *miR-30c* in primary lung tumors and their adjacent normal tissues, as found with the Wilcoxon test.
(TIF)

Figure S7 *miR-30c* directly targets Vimentin and Fibronectin. (A) The putative *miR-30c*-binding sites in the Vimentin or Fibronectin 3′UTR. nt, nucleotides. BS, Binding Sites. (B) Immunoblot analysis for Vimentin or Fibronectin in *miR-30c*-transfected cells, H1299 and A549. *miR-20a*, another negative control. (C) qRT-PCR for Vimentin mRNA in presence of *miR-30c*. * *P*<0.01 by Student's *t*-test.
(TIF)

Figure S8 The inverse correlation between *miR-30c* and Vimentin in primary lung tissues (A) and their adjacent normal tissues (B).
(TIF)

Figure S9 miR-30c directly targets MTDH and HMGA2. (A, B) The putative miR-30c-binding sites in the MTDH and HMGA2 3'UTRs.
(TIF)

Figure S10 The mRNA levels of MTDH (A) or HMGA2 (B) in primary lung tissues and their adjacent normal tissues.
(TIF)

Figure S11 The expression of Fhit protein in MTDH or HMGA2 knockdown A549 cells was measured by Immunoblot analysis.
(TIF)

Figure S12 Expression pattern of Fhit, *miR-30c*, MTDH and HMGA2 in primary lung tissues (P) and their matched metastatic lymph node tissues (M.L), measured by quantitative real time PCR. * *P*<0.05 and ** *P*<0.0001 by Student's *t*-test.
(TIF)

Table S1 A profiling data for miRNAs modulated by Fhit. *P*<0.05, Fhit *vs* Control >1.2 fold change.
(DOCX)

Text S1 List of primers used in the study.
(DOCX)
