# Supplementary figures and images for: FHIT Suppresses Epithelial-Mesenchymal Transition (EMT) and Metastasis in Lung Cancer through Modulation of MicroRNAs
Source: PLoS Genet. 2014 Oct 23;10(10):e1004652. doi: 10.1371/journal.pgen.1004652 (PMC4207614; doi:10.1371/journal.pgen.1004652)

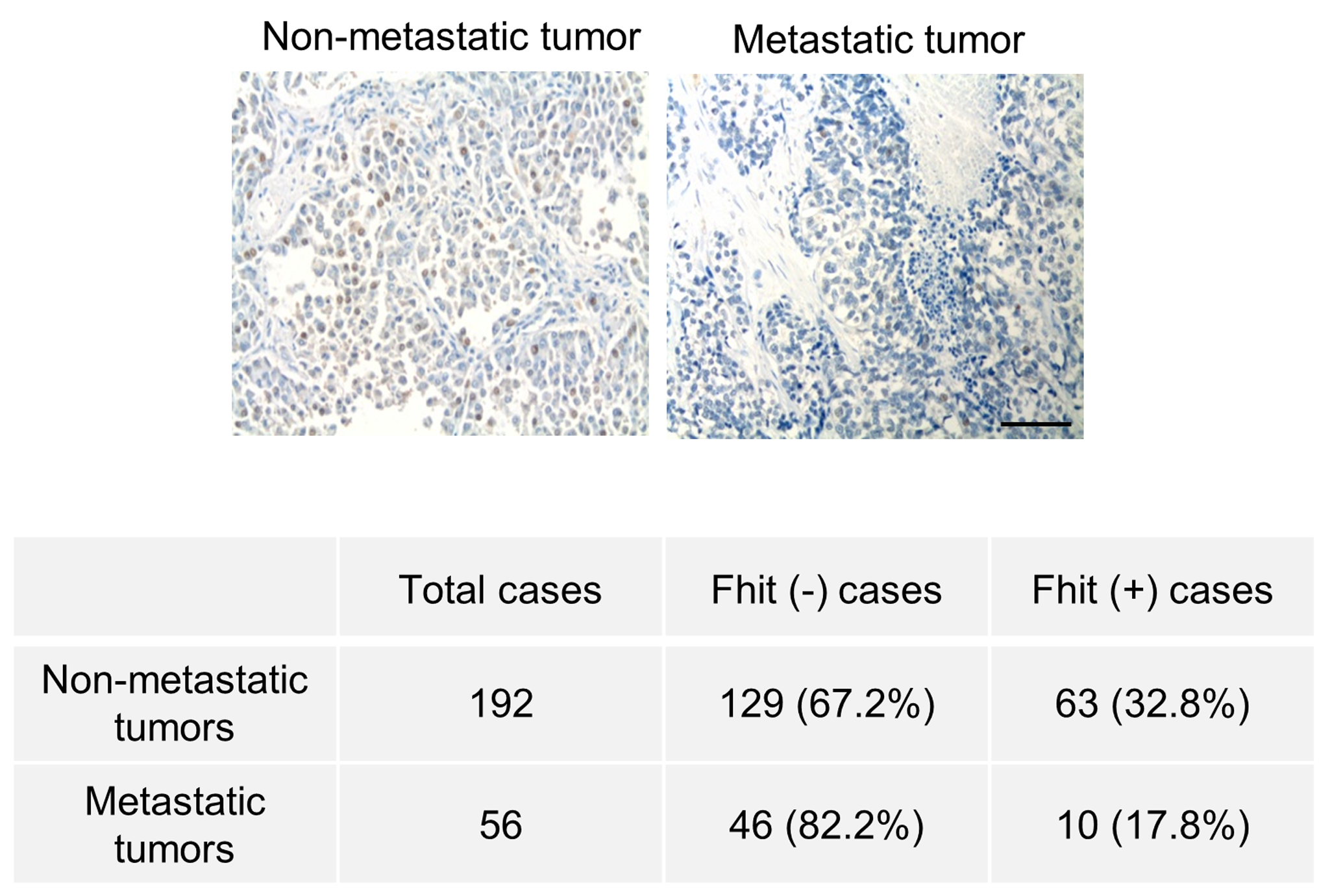

Supplement: Figure S1 — Immunohistochemistry assay to detect Fhit expression in Non-metastatic and Metastatic tumors. (TIF) [file pgen.1004652.s001.tif]

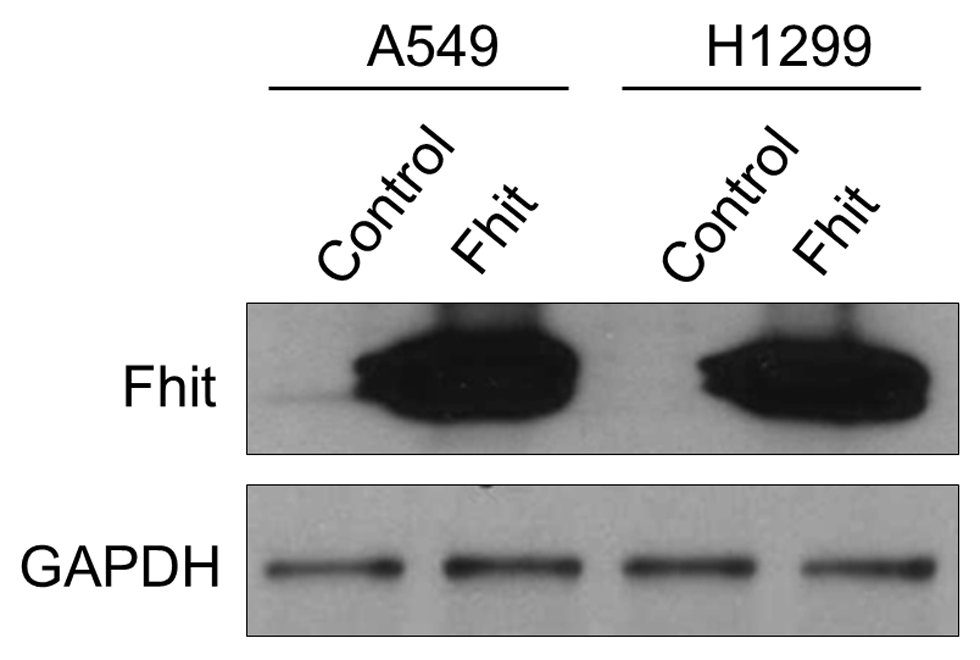

Supplement: Figure S2 — The expression of Fhit protein in Fhit-overexpressing cells, A549 and H1299 were measured by western blotting. (TIF) [file pgen.1004652.s002.tif]

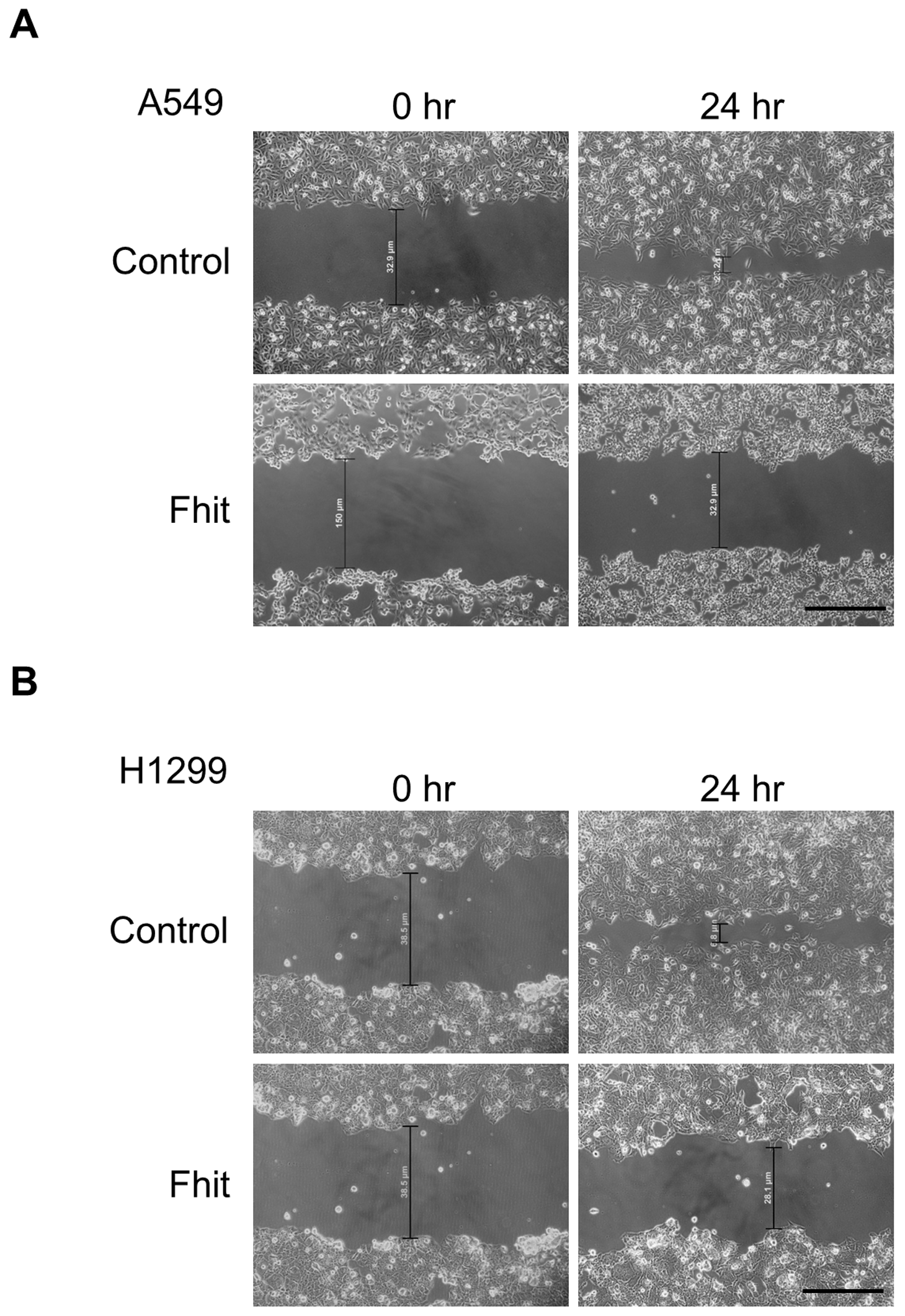

Supplement: Figure S3 — Representative photographs of scratched areas of the confluent monolayer of A549 (A) or H1299 cells (B) stably expressing Fhit or control vector at 0 h and 24 h after wounding with a pipet tip. Scale bar, 500 µm. (TIF) [file pgen.1004652.s003.tif]

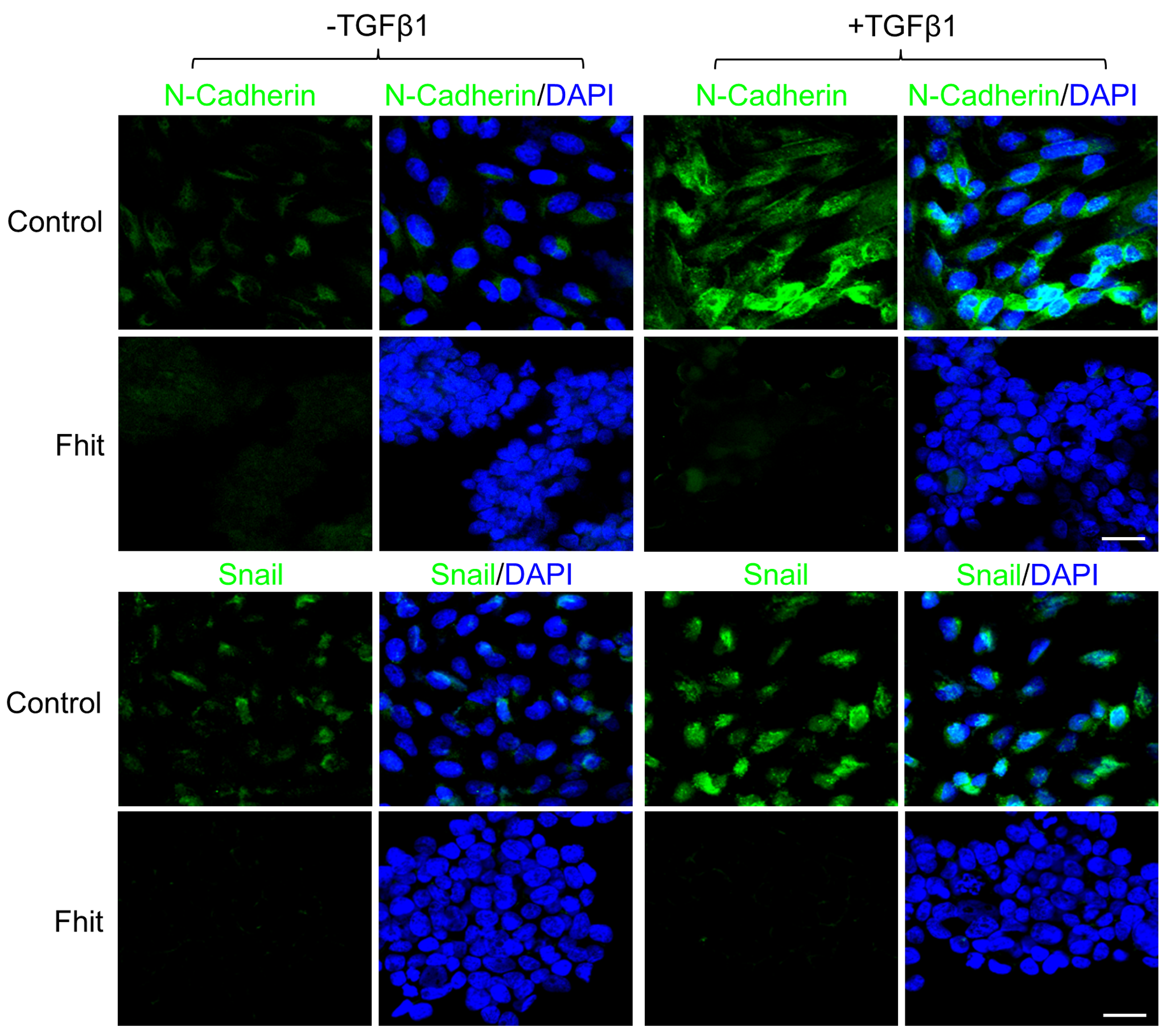

Supplement: Figure S4 — Immunofluorescence assay for N-cadherin in Fhit-expressing or control A549 cells treated with TGF-β-1. Scale bar, 20 µm. (TIF) [file pgen.1004652.s004.tif]

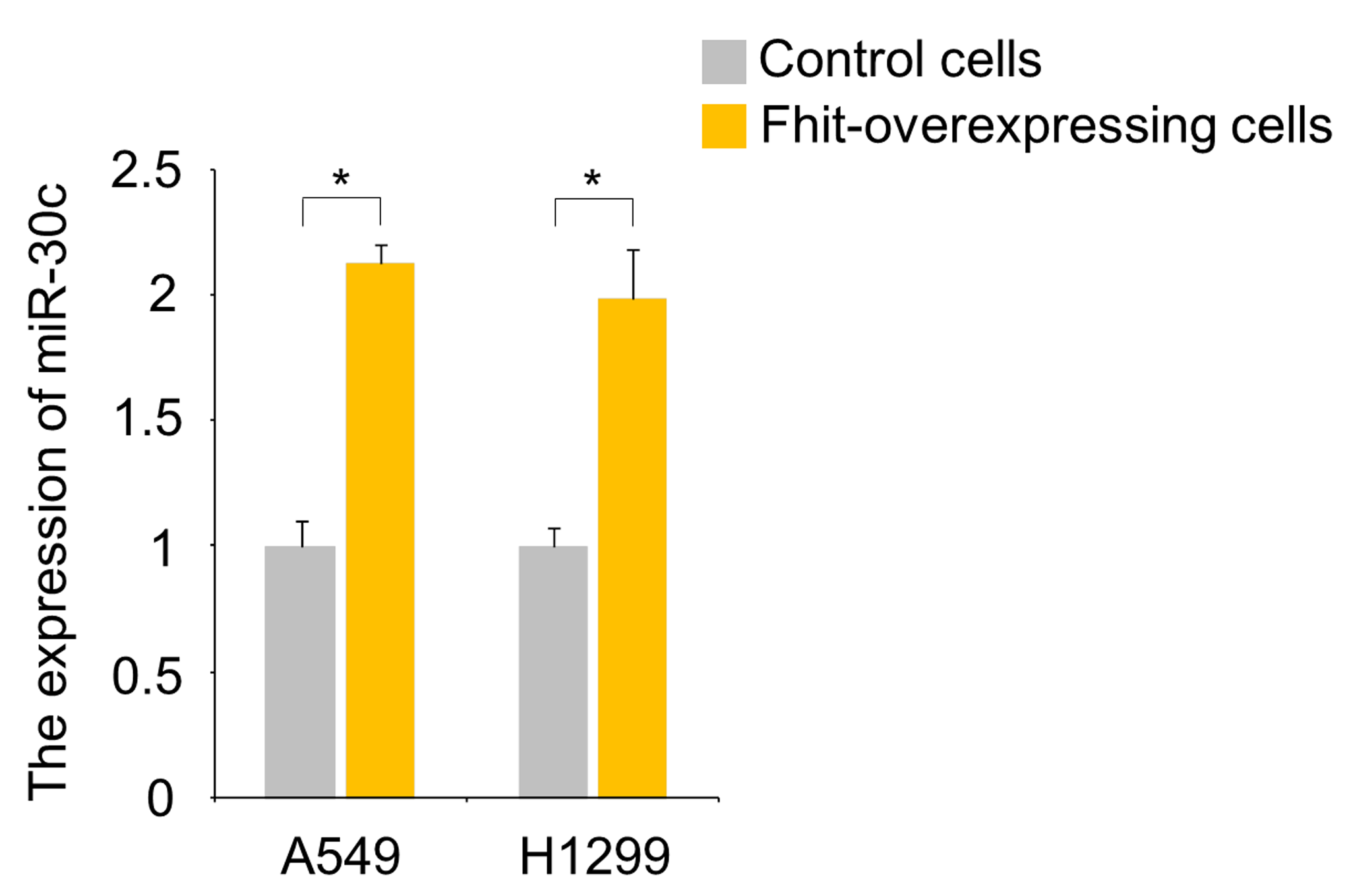

Supplement: Figure S5 — The expression levels of miR-30c in miR-30c-overexpressing cells, A549 and H1299. * P<0.05 by Student's t-test. (TIF) [file pgen.1004652.s005.tif]

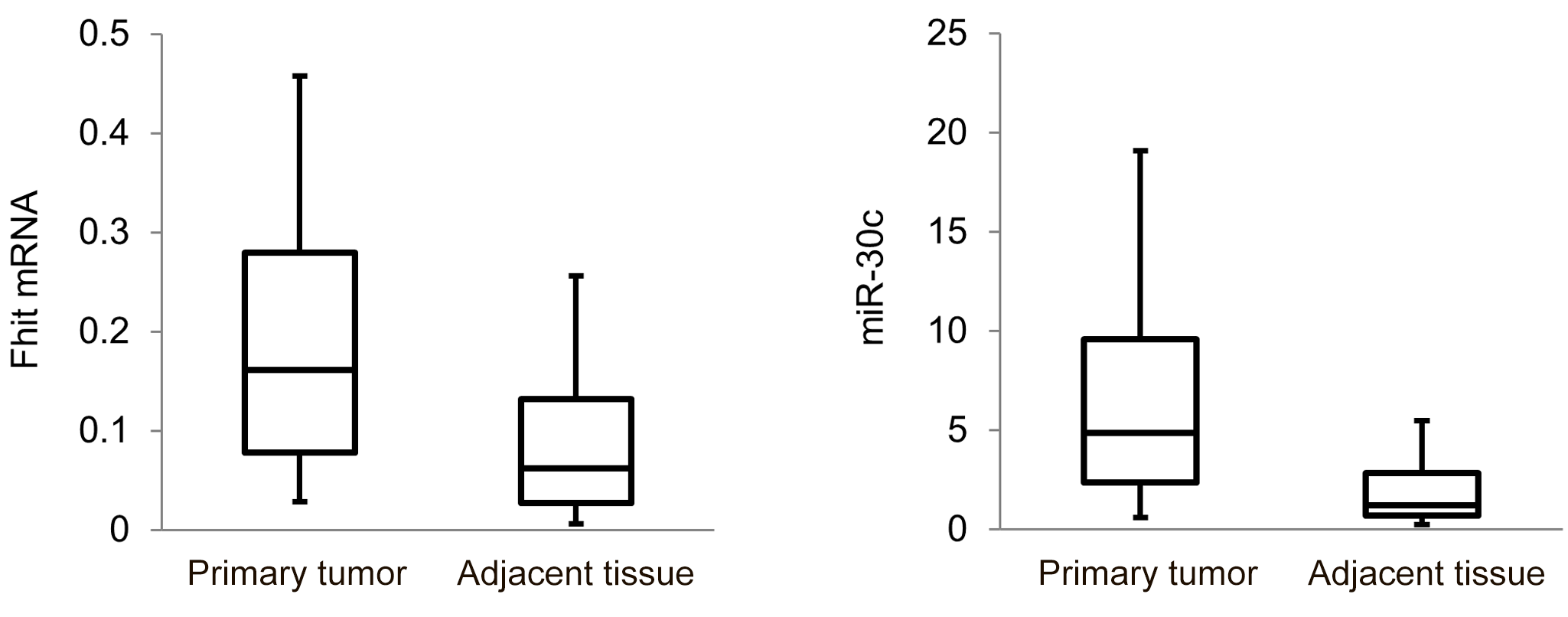

Supplement: Figure S6 — The different expression levels of Fhit and miR-30c in primary lung tumors and their adjacent normal tissues, as found with the Wilcoxon test. (TIF) [file pgen.1004652.s006.tif]

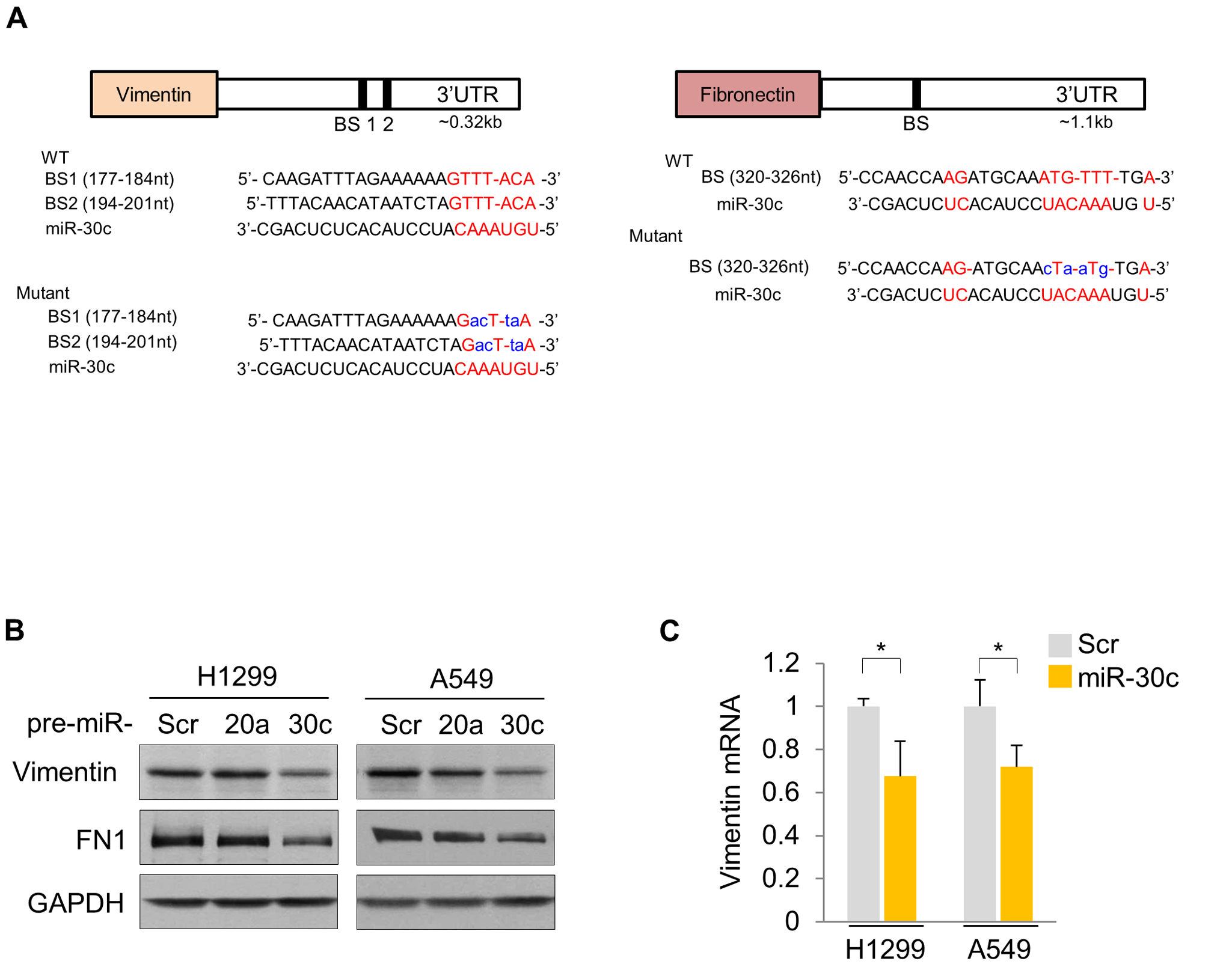

Supplement: Figure S7 — miR-30c directly targets Vimentin and Fibronectin. (A) The putative miR-30c-binding sites in the Vimentin or Fibronectin 3′UTR. nt, nucleotides. BS, Binding Sites. (B) Immunoblot analysis for Vimentin or Fibronectin in miR-30c-transfected cells, H1299 and A549. miR-20a, another negative control. (C) qRT-PCR for Vimentin mRNA in presence of miR-30c. * P<0.01 by Student's t-test. (TIF) [file pgen.1004652.s007.tif]

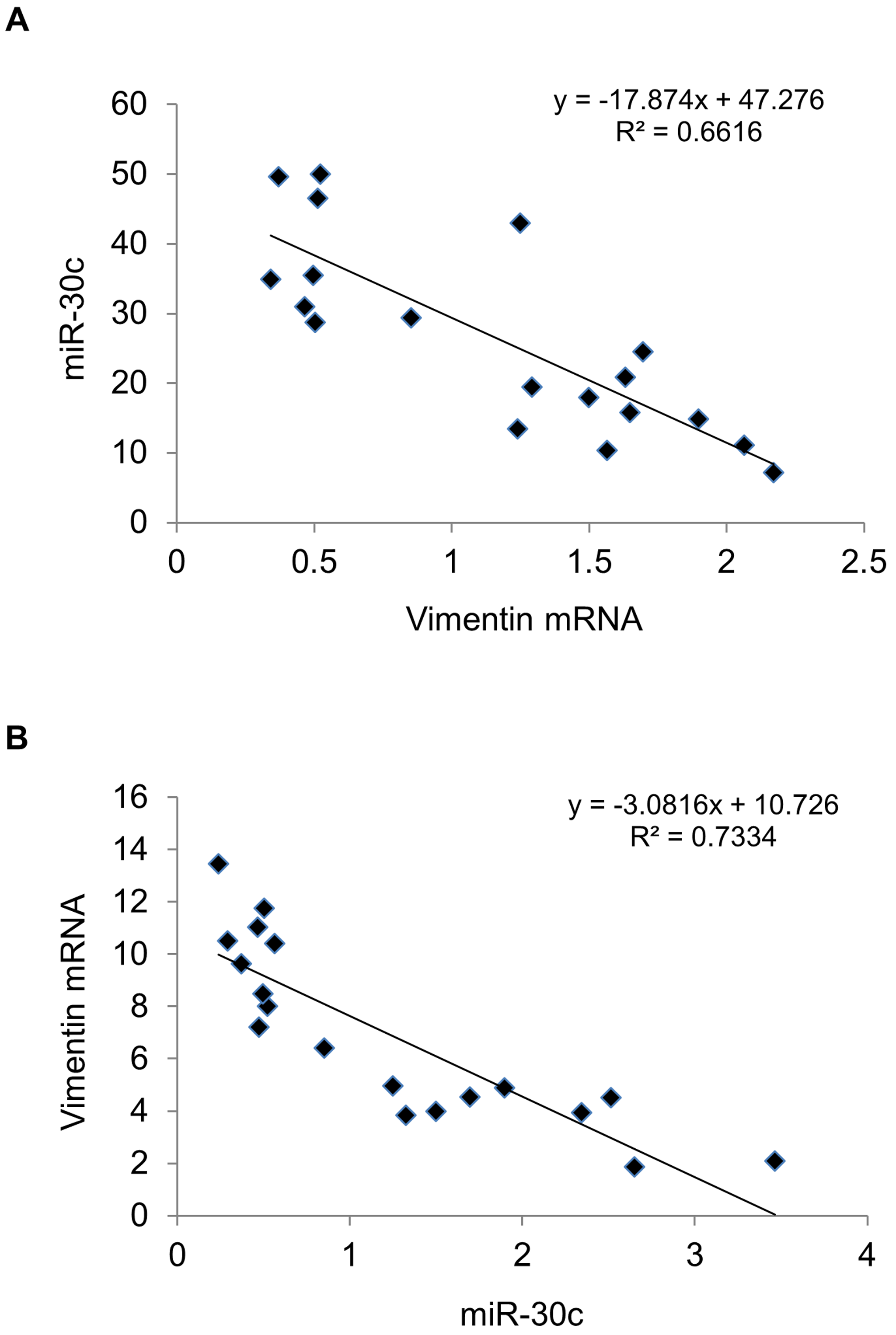

Supplement: Figure S8 — The inverse correlation between miR-30c and Vimentin in primary lung tissues (A) and their adjacent normal tissues (B). (TIF) [file pgen.1004652.s008.tif]

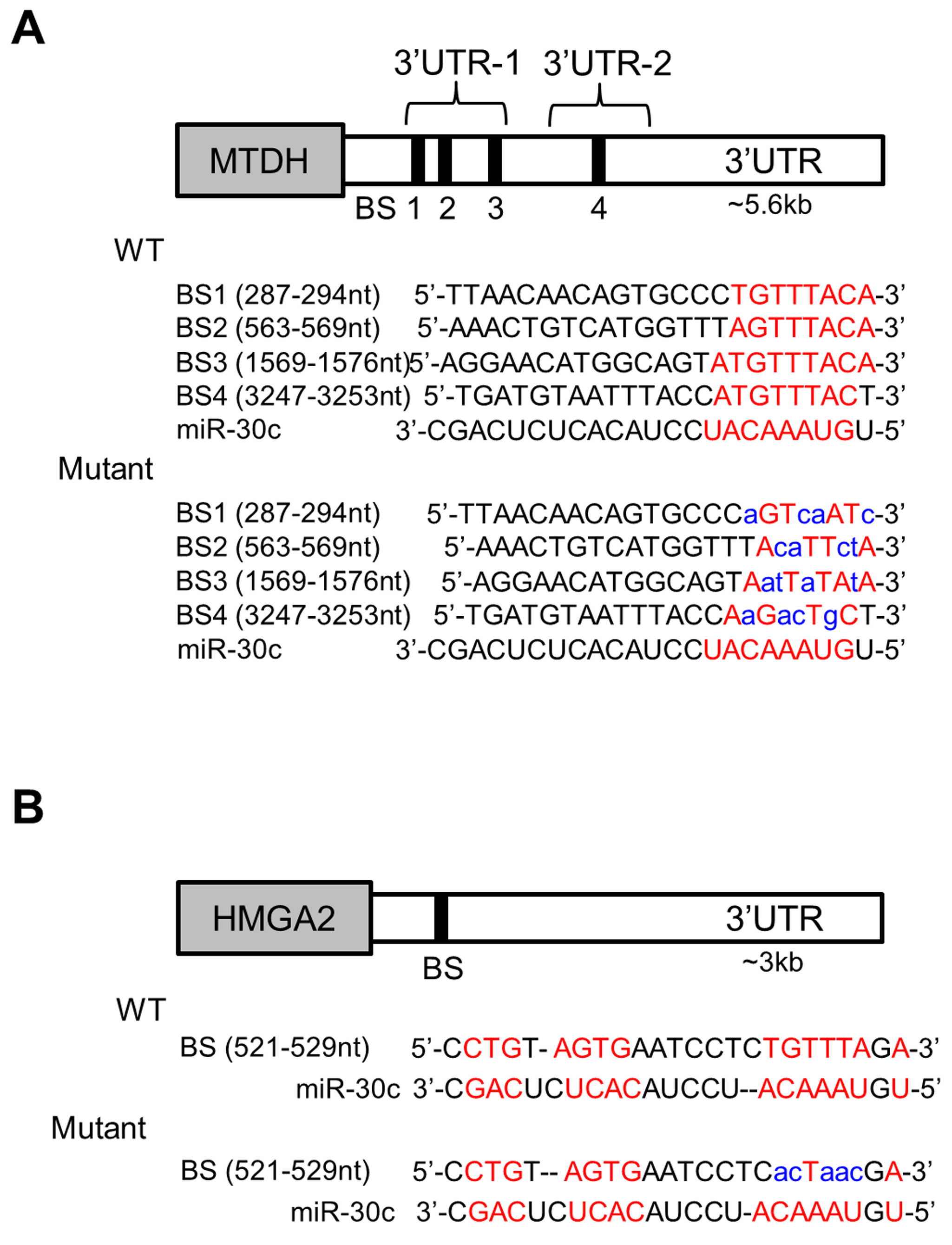

Supplement: Figure S9 — miR-30c directly targets MTDH and HMGA2. (A, B) The putative miR-30c-binding sites in the MTDH and HMGA2 3'UTRs. (TIF) [file pgen.1004652.s009.tif]

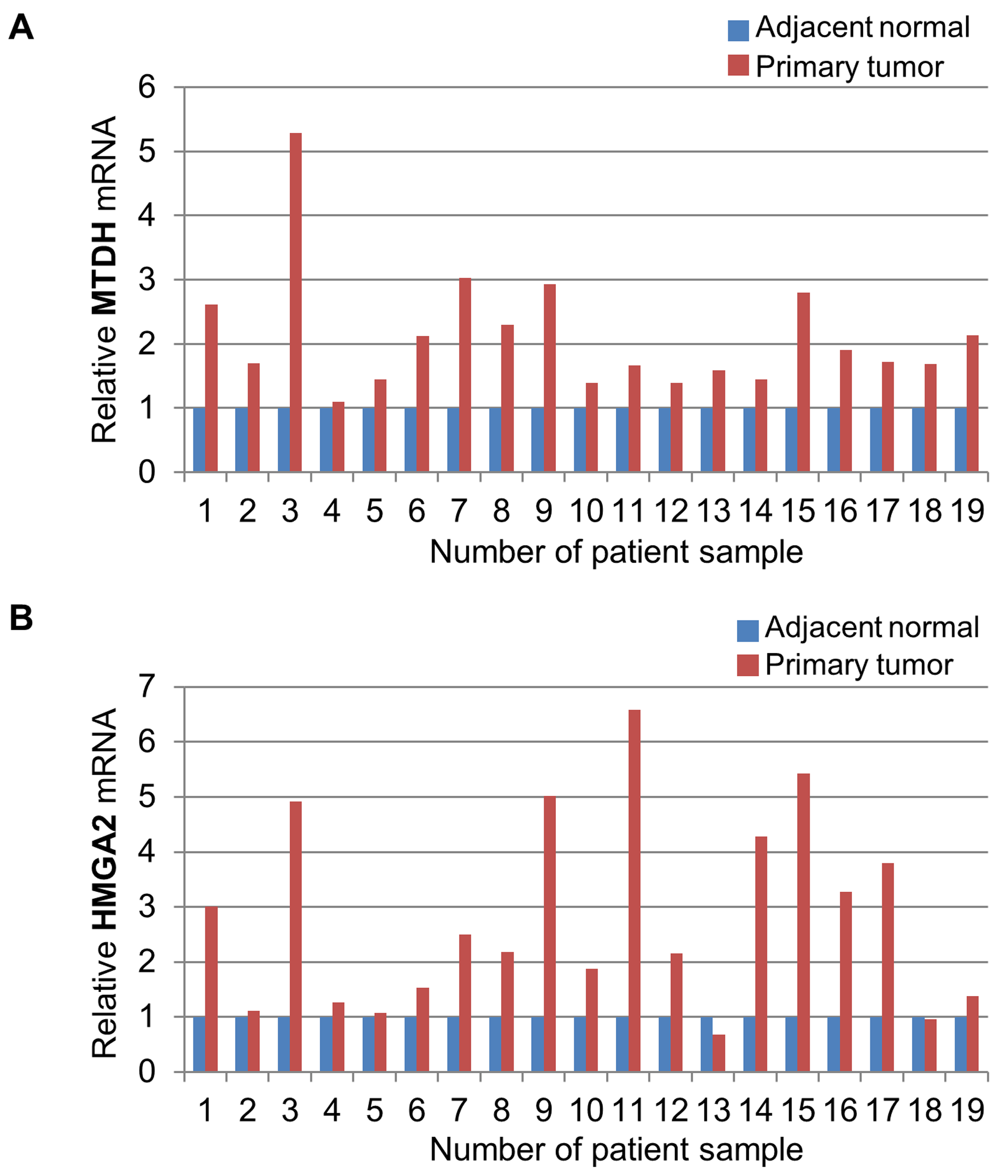

Supplement: Figure S10 — The mRNA levels of MTDH (A) or HMGA2 (B) in primary lung tissues and their adjacent normal tissues. (TIF) [file pgen.1004652.s010.tif]

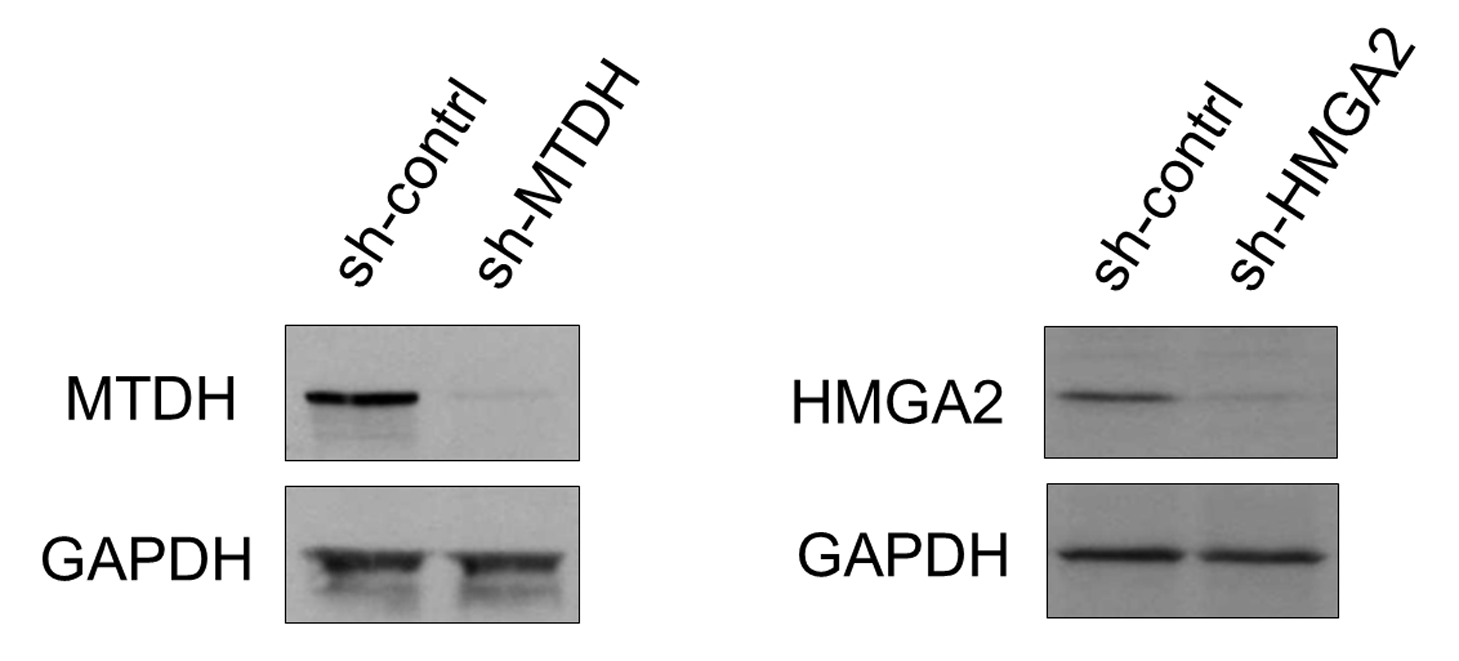

Supplement: Figure S11 — The expression of Fhit protein in MTDH or HMGA2 knockdown A549 cells was measured by Immunoblot analysis. (TIF) [file pgen.1004652.s011.tif]

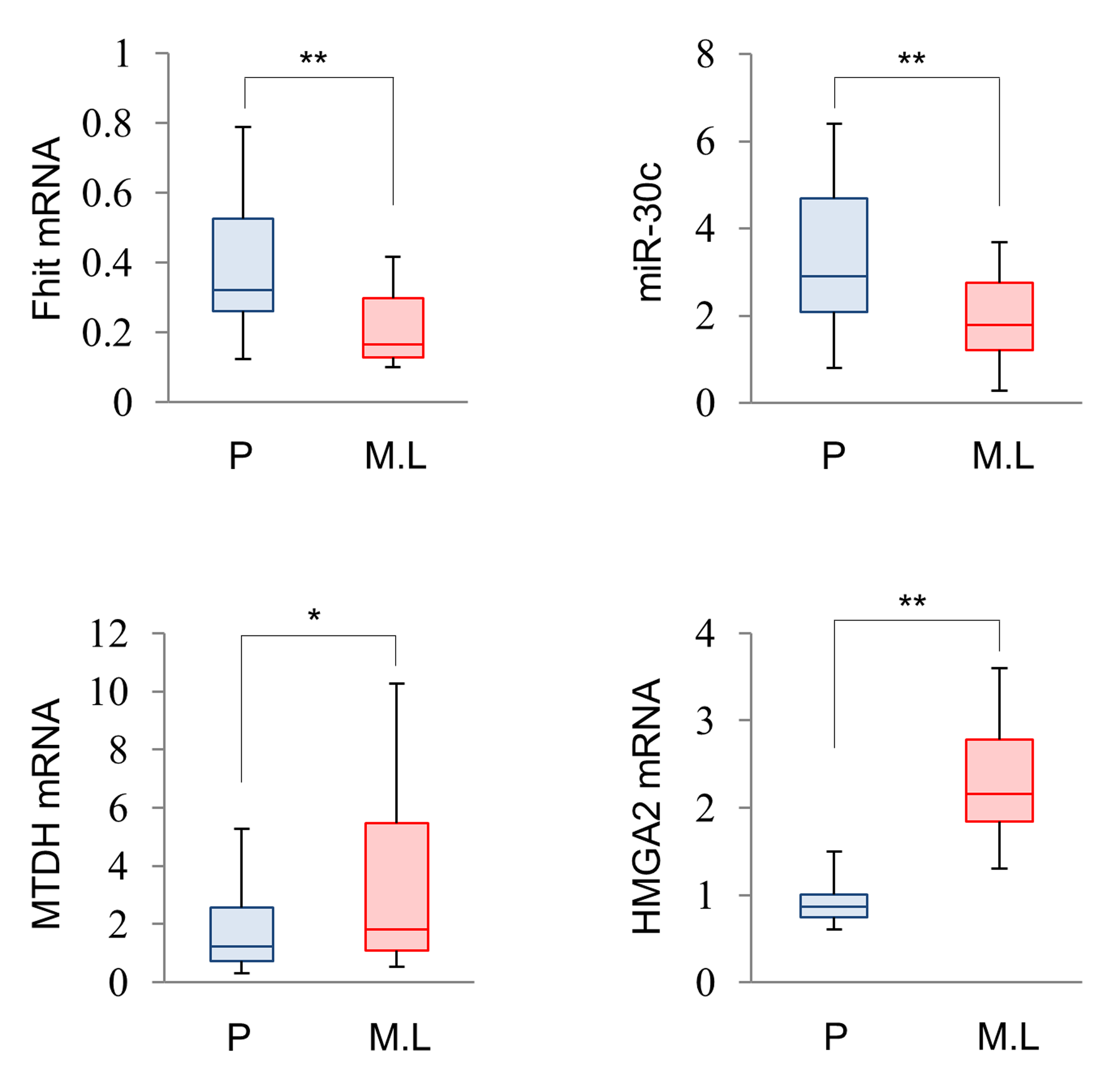

Supplement: Figure S12 — Expression pattern of Fhit, miR-30c, MTDH and HMGA2 in primary lung tissues (P) and their matched metastatic lymph node tissues (M.L), measured by quantitative real time PCR. * P<0.05 and ** P<0.0001 by Student's t-test. (TIF) [file pgen.1004652.s012.tif]
