# Supplementary material for: FHIT Suppresses Epithelial-Mesenchymal Transition (EMT) and Metastasis in Lung Cancer through Modulation of MicroRNAs
Source: PLoS Genet. 2014 Oct 23;10(10):e1004652. doi: 10.1371/journal.pgen.1004652 (PMC4207614; doi:10.1371/journal.pgen.1004652)
Supplement: Table S1 — A profiling data for miRNAs modulated by Fhit. P<0.05, Fhit vs Control >1.2 fold change. (DOCX) [file pgen.1004652.s013.docx]

| Detector | Mean.Fhit | Mean.Control | SD.Fhit | SD.Control | P.val | FoldChange.  Fhit/Control | FoldChange.  Fhit/Control |
| --- | --- | --- | --- | --- | --- | --- | --- |
| hsa-miR-9 | 268.9881562 | 165.4602242 | 10.17472693 | 8.13836808 | 5.931E-06 | 1.625696795 | 1.625696795 |
| hsa-miR-26a | 1506.106165 | 2550.276556 | 106.901505 | 61.8068752 | 1.823E-05 | 0.59056582 | -1.693291359 |
| hsa-miR-1308 | 246.7431415 | 152.7678592 | 11.56930212 | 11.45808184 | 2.5438E-05 | 1.615150875 | 1.615150875 |
| hsa-miR-30c | 356.1107587 | 156.1951106 | 10.36907147 | 10.27015849 | 2.9044E-05 | 2.27990977 | 2.27990977 |
| hsa-let-7a | 3273.078482 | 4572.652213 | 148.61693 | 283.4497262 | 5.9215E-05 | 0.71579432 | -1.397049365 |
| hsa-miR-18a | 130.8846093 | 95.11297979 | 3.227641733 | 5.777976704 | 0.00016822 | 1.376096192 | 1.376096192 |
| hsa-miR-191 | 246.7630789 | 328.8277847 | 17.3148476 | 13.52550963 | 0.00038703 | 0.750432568 | -1.332564766 |
| hsa-miR-20a | 1977.374341 | 1287.649326 | 136.1652887 | 146.3315948 | 0.00046765 | 1.535646625 | 1.535646625 |
| hsa-miR-222 | 160.7621129 | 95.46458459 | 14.31982881 | 9.211935918 | 0.00053951 | 1.683997407 | 1.683997407 |
| hsa-miR-30a | 128.6750213 | 83.90987007 | 8.642566704 | 7.318103633 | 0.00094483 | 1.533490889 | 1.533490889 |
| hsa-let-7d | 137.15772 | 242.8984756 | 9.142018406 | 22.2959533 | 0.00094975 | 0.564670979 | -1.770942792 |
| hsa-miR-92a | 3202.99971 | 2294.15671 | 111.6152213 | 221.6060586 | 0.00123141 | 1.396155588 | 1.396155588 |
| hsa-miR-361-5p | 198.256603 | 256.2753144 | 15.90570509 | 11.34433218 | 0.00145644 | 0.773607881 | -1.292644535 |
| hsa-miR-125a-5p | 478.7342673 | 562.5405189 | 21.41620917 | 24.37831463 | 0.00218943 | 0.85102184 | -1.175057975 |
| hsa-miR-125b | 1480.327749 | 3135.986022 | 228.9939372 | 471.6763881 | 0.00243183 | 0.472045391 | -2.118440342 |
| hsa-miR-148b | 169.4529638 | 71.51568122 | 52.13669508 | 20.33260951 | 0.02602511 | 2.369451859 | 2.369451859 |
| hsa-let-7f | 412.2223431 | 636.3641955 | 34.80167424 | 67.0317905 | 0.00274492 | 0.647777399 | -1.543740183 |
| hsa-miR-29c | 106.7577851 | 61.49309679 | 6.866373862 | 7.837317705 | 0.00299112 | 1.736093816 | 1.736093816 |
| hsa-miR-100 | 2898.159847 | 5744.463344 | 128.7662663 | 700.1261098 | 0.00320651 | 0.504513594 | -1.982107147 |
| hsa-miR-15a | 1085.232866 | 856.9710438 | 43.03323527 | 73.22790709 | 0.00328985 | 1.266358851 | 1.266358851 |
| hsa-miR-221 | 4040.351952 | 3327.582369 | 100.6651466 | 220.6832717 | 0.00359886 | 1.214200432 | 1.214200432 |
| hsa-miR-23a | 817.3901897 | 640.0436056 | 65.95753867 | 37.74557981 | 0.00617206 | 1.277085159 | 1.277085159 |
| hsa-miR-137 | 151.1862507 | 197.5736426 | 3.462668871 | 15.48311945 | 0.00760912 | 0.765214675 | -1.306822821 |
| hsa-miR-374b | 259.5322477 | 330.124422 | 11.06477194 | 26.42106736 | 0.00777432 | 0.786164944 | -1.271997699 |
| hsa-miR-374a | 391.2331922 | 525.7925392 | 25.79021378 | 53.92654905 | 0.00911561 | 0.744082814 | -1.343936429 |
| hsa-miR-19b | 963.6835866 | 758.7811493 | 85.58842697 | 65.37040427 | 0.01011238 | 1.270041549 | 1.270041549 |
| hsa-miR-103 | 1349.721774 | 1046.121072 | 48.39187875 | 125.8491851 | 0.01165713 | 1.290215646 | 1.290215646 |
| hsa-miR-484 | 160.415599 | 116.2108003 | 14.02975532 | 20.5924515 | 0.01493307 | 1.380384599 | 1.380384599 |
| hsa-miR-106a | 107.4637927 | 89.32237037 | 7.791139776 | 7.914415956 | 0.01710427 | 1.203100547 | 1.203100547 |
| hsa-miR-1260 | 1689.104135 | 1227.153938 | 180.4801883 | 222.9938038 | 0.01923966 | 1.3764403 | 1.3764403 |
| hsa-miR-301a | 135.6520579 | 97.81420798 | 18.79673854 | 9.164248603 | 0.01938224 | 1.386833883 | 1.386833883 |
| hsa-miR-151-5p | 134.6986779 | 104.5653104 | 14.67975465 | 11.82375607 | 0.01984685 | 1.288177479 | 1.288177479 |
| hsa-miR-21 | 3457.194968 | 2711.41551 | 333.7765516 | 340.8886313 | 0.02043021 | 1.275051705 | 1.275051705 |
| hsa-let-7e | 124.6963573 | 160.2797534 | 10.24449487 | 21.08237087 | 0.0346477 | 0.777991947 | -1.285360349 |
| hsa-miR-542-3p | 102.7429016 | 76.78170189 | 6.556670529 | 15.49356561 | 0.03620936 | 1.338117013 | 1.338117013 |
