# Supplementary material for: FHIT Suppresses Epithelial-Mesenchymal Transition (EMT) and Metastasis in Lung Cancer through Modulation of MicroRNAs
Source: PLoS Genet. 2014 Oct 23;10(10):e1004652. doi: 10.1371/journal.pgen.1004652 (PMC4207614; doi:10.1371/journal.pgen.1004652)
Supplement: Text S1 — List of primers used in the study. (DOCX) [file pgen.1004652.s014.docx]

**List of primers used for wild type 3’UTR luciferase constructs.**

| MTDH 3'UTR-1 (F) | 5’-atttctagaTTATGAAATTAAGAGGTCAG-3’ |
| --- | --- |
| MTDH 3'UTR-1 (R) | 5’-atttctagaGCTATCCATTTGCTGAATTT-3’ |
| MTDH 3'UTR-2 (F) | 5’-atttctagaCAGAACTAGTAGGGAAATAA-3’ |
| MTDH 3'UTR-2 (R) | '5’-atctctagaTTGTGCAGTGAGTTTACAGG-3’ |
| HMGA2 3'UTR (F) | 5’-atttctagaTGGGGAGAAATCACATAACC-3’ |
| HMGA2 3'UTR (R) | 5’-atctctagaTCCTGCTGATATGTGTGATA-3’ |
| FN1 3'UTR (F) | 5’-atttctagaTCCAATCCAGAGGAACAAGC-3’ |
| FN1 3'UTR (R) | 5’-acttctagaCTTAGTCATTTTTATTTCCC-3’ |
| Vimentin 3'UTR (F) | 5’-atttctagaGCACACACTCAGTGCAGCAATATA-3’ |
| Vimentin 3'UTR (R) | 5’-acttctagaGGTTGGATACTTGCTGGAAAAAA-3’ |

|  |
| --- |

**List of primers used for mutant 3’UTR luciferase constructs.**

| MTDH (mut-F1) | 5'-CTGGTTTAACAACAGTGCCCAGTCAATCACAGATTGTGCCCTATCTCA-3 |
| --- | --- |
| MTDH (mut-R1) | 5'-TGAGATAGGGCACAATCTGTGATTGACTGGGCACTGTTGTTAAACCAG-3 |
| MTDH (mut-F2) | 5'-AACTAAACTGTCATGGTTTACATTCTAATTTTTAAAAAGTTCTTAAAA-3' |
| MTDH (mut-R2) | 5'-TTTTAAGAACTTTTTAAAAATTAGAATGTAAACCATGACAGTTTAGTT-3' |
| MTDH (mut-F3) | 5'-GTTTCAGGAACATGGCAGTAATTATATATGTCAGAAGTTTTGTTTAAT-3' |
| MTDH (mut-R3) | 5'-ATTAAACAAAACTTCTGACATATATAATTACTGCCATGTTCCTGAAAC-3 |
| MTDH (mut-F4) | 5'-GTGATTGATGTAATTTACCAAGACTGCTTTATGCATGCATTTTATTG-3' |
| MTDH (mut-R4) | 5'-CAATAAAATGCATGCATAAAGCAGTCTTGGTAAATTACATCAATCAC-3' |
| HMGA2 (mut-F) | 5’-ACTCCCTGTAGTGAATCCTCACTAACCAACACCAAAGATAAGGACTAG-3’ |
| HMGA2 (mut-R) | 5’-CTAGTCCTTATCTTTGGTGTTGGTTAGTGAGGATTCACTACAGGGAGT-3’ |
| FN1 (mut-F) | 5’-TGCAGCCAACCAAGATGCAACTAATGTGAAATGATATGACCAAAAT-3’ |
| FN1 (mut-R) | 5’-ATTTTGGTCATATCATTTCACATTAGTTGCATCTTGGTTGGCTGCA-3’ |
| Vimentin(mut-F) | 5’-TTAGAAAAAAGACTTAAACATAATCTAGACTTAAGAAAAA-3’ |
| Vimentin(mut-R) | 5’-TTTTTCTTAAGTCTAGATTATGTTTAAGTCTTTTTTCTAA-3’ |
